# Weight regain and cardiometabolic effects after withdrawal of semaglutide: The STEP 1 trial extension

**DOI:** 10.1111/dom.14725

**Published:** 2022-05-19

**Authors:** John P. H. Wilding, Rachel L. Batterham, Melanie Davies, Luc F. Van Gaal, Kristian Kandler, Katerina Konakli, Ildiko Lingvay, Barbara M. McGowan, Tugce Kalayci Oral, Julio Rosenstock, Thomas A. Wadden, Sean Wharton, Koutaro Yokote, Robert F. Kushner, STEP 1 Study Group

**Affiliations:** ^1^ Department of Cardiovascular and Metabolic Medicine, Institute of Life Course and Medical Sciences University of Liverpool Liverpool UK; ^2^ University College London Centre for Obesity Research, Division of Medicine University College London London UK; ^3^ National Institute of Health Research UCLH Biomedical Research Centre London UK; ^4^ Centre for Weight Management and Metabolic Surgery, University College London Hospital London UK; ^5^ Diabetes Research Centre University of Leicester Leicester UK; ^6^ NIHR Leicester Biomedical Research Centre Leicester UK; ^7^ Department of Endocrinology, Diabetology and Metabolism Antwerp University Hospital, University of Antwerp Antwerp Belgium; ^8^ Novo Nordisk A/S Søborg Denmark; ^9^ Departments of Internal Medicine/Endocrinology and Department of Population and Data Sciences University of Texas Southwestern Medical Center Dallas Texas USA; ^10^ Department of Diabetes and Endocrinology Guy's and St Thomas' NHS Foundation Trust London UK; ^11^ Dallas Diabetes Research Center at Medical City Dallas Texas USA; ^12^ Department of Psychiatry, Perelman School of Medicine University of Pennsylvania Philadelphia Pennsylvania USA; ^13^ York University, McMaster University and Wharton Weight Management Clinic Toronto Ontario Canada; ^14^ Department of Endocrinology, Hematology and Gerontology, Graduate School of Medicine, Chiba University and Department of Diabetes, Metabolism and Endocrinology Chiba University Hospital Chiba Japan; ^15^ Division of Endocrinology, Feinberg School of Medicine Northwestern University Chicago Illinois USA

**Keywords:** antiobesity drug, clinical trial, GLP‐1 analogue, obesity therapy, phase III study, weight control

## Abstract

**Aim:**

To explore changes in body weight and cardiometabolic risk factors after treatment withdrawal in the STEP 1 trial extension.

**Materials and Methods:**

STEP 1 (NCT03548935) randomized 1961 adults with a body mass index ≥ 30 kg/m^2^ (or ≥ 27 kg/m^2^ with ≥ 1 weight‐related co‐morbidity) without diabetes to 68 weeks of once‐weekly subcutaneous semaglutide 2.4 mg (including 16 weeks of dose escalation) or placebo, as an adjunct to lifestyle intervention. At week 68, treatments (including lifestyle intervention) were discontinued. An off‐treatment extension assessed for a further year a representative subset of participants who had completed 68 weeks of treatment. This subset comprised all eligible participants from any site in Canada, Germany and the UK, and sites in the United States and Japan with the highest main phase recruitment. All analyses in the extension were exploratory.

**Results:**

Extension analyses included 327 participants. From week 0 to week 68, mean weight loss was 17.3% (SD: 9.3%) with semaglutide and 2.0% (SD: 6.1%) with placebo. Following treatment withdrawal, semaglutide and placebo participants regained 11.6 (SD: 7.7) and 1.9 (SD: 4.8) percentage points of lost weight, respectively, by week 120, resulting in net losses of 5.6% (SD: 8.9%) and 0.1% (SD: 5.8%), respectively, from week 0 to week 120. Cardiometabolic improvements seen from week 0 to week 68 with semaglutide reverted towards baseline at week 120 for most variables.

**Conclusions:**

One year after withdrawal of once‐weekly subcutaneous semaglutide 2.4 mg and lifestyle intervention, participants regained two‐thirds of their prior weight loss, with similar changes in cardiometabolic variables. Findings confirm the chronicity of obesity and suggest ongoing treatment is required to maintain improvements in weight and health.

## 1 INTRODUCTION

Obesity is a highly prevalent, complex, chronic disease^1^, ^2^, ^3^, ^4^ associated with cardiometabolic complications, including type 2 diabetes, hypertension, dyslipidaemia and cardiovascular disease.^4^, ^5^, ^6^ Obesity also leads to a wide range of other health problems, is associated with substantial socioeconomic burden and was estimated to cause 5 million deaths globally in 2019.^2^, ^3^, ^4^, ^7^

Weight loss activates compensatory biological changes that prevent the maintenance of long‐term weight loss^8^, ^9^; weight regain is common.^8^, ^10^ In people with obesity, pharmacotherapy is indicated as an adjunct to lifestyle intervention for chronic weight management and can help with achieving and maintaining weight loss.^2^, ^11^, ^12^ Long‐term obesity pharmacotherapy may be required for weight maintenance, as cessation of pharmacological treatment is frequently followed by weight regain, even with continued lifestyle intervention.^13^, ^14^, ^15^

The present observational study examined changes in body weight and cardiometabolic risk factors over 52 weeks following cessation of once‐weekly subcutaneous (s.c.) semaglutide 2.4 mg (or placebo) and lifestyle intervention in participants who had completed 68 weeks of initial treatment in the randomized, placebo‐controlled Semaglutide Treatment Effect in People with obesity (STEP) 1 trial.^16^ Semaglutide is a glucagon‐like peptide‐1 (GLP‐1) analogue recently approved by the US Food and Drug Administration, Health Canada and the UK Medicines and Healthcare products Regulatory Agency for chronic weight management, as an adjunct to lifestyle intervention, at a once‐weekly s.c. dose of 2.4 mg in adults with overweight (with ≥ 1 weight‐related condition) or obesity.^17^, ^18^, ^19^ The efficacy and safety of semaglutide 2.4 mg for weight management have been investigated in the global phase III STEP programme.^20^ In the main phase of the STEP 1 trial, 68 weeks of treatment with semaglutide plus lifestyle intervention in adults with overweight/obesity produced clinically meaningful reductions in body weight, as well as improvements in cardiometabolic risk factors, and physical functioning.^16^

A prior trial (STEP 4) assessed the effects of semaglutide withdrawal, with participants initially receiving 20 weeks of semaglutide 2.4 mg treatment during a run‐in period, followed by randomization to continued semaglutide treatment or withdrawal (switch to placebo) for an additional 48 weeks, with lifestyle intervention throughout.^13^ The present STEP 1 extension study complements STEP 4 by exploring post‐treatment changes in body weight and cardiometabolic risk factors following a longer (68‐week) initial treatment period with semaglutide or placebo, and in the absence of active lifestyle intervention support during the 1‐year off‐treatment follow‐up period.

## 2 MATERIALS AND METHODS

### 2.1 Trial design and participants

STEP 1 (NCT03548935) was a randomized, double‐blind, placebo‐controlled trial conducted at 129 sites across 16 countries. The design and eligibility criteria have previously been published.^16^ Participants were adults (aged ≥ 18 years) with a body mass index (BMI) of 30 kg/m^2^ or higher, or of 27 kg/m^2^ or higher with at least one weight‐related co‐morbidity (hypertension, dyslipidaemia, obstructive sleep apnoea or cardiovascular disease), and a history of at least one self‐reported unsuccessful dietary effort to lose weight. Key exclusion criteria were type 1 or 2 diabetes and obesity pharmacotherapy 90 days or less before enrolment. Participants were randomized to 68 weeks of treatment with once‐weekly s.c. semaglutide 2.4 mg (n = 1306) or placebo (n = 655) (2:1), plus lifestyle intervention. The lifestyle intervention consisted of counselling every 4 weeks on diet (500 kcal deficit per day relative to total estimated energy expenditure at randomization) and physical activity (150 minutes per week). Semaglutide was initiated at 0.25 mg, with escalation every 4 weeks until the 2.4 mg target dose was reached (Figure S1). At week 68 (the end of the treatment period), participants were withdrawn from treatment (including lifestyle intervention) and followed for 7 weeks until week 75.

The STEP 1 extension followed a subset of participants for an additional 45 weeks (a total of 52 weeks off‐treatment) until the end‐of‐trial visit at week 120 (Figure S1). The extension was offered in five selected countries (Canada, Germany, Japan, the UK and the United States) that were representative of the global trial population and aimed to include approximately 300 participants. In Canada, Germany and the UK, all sites with subjects interested in participating in the extension were included (Canada: six sites; Germany: 13 sites; UK: 10 sites). In the United States and Japan, the sites included in the extension (United States: five sites; Japan: three sites) were objectively selected based on those with the highest recruitment in the STEP 1 main phase (using an assumed on‐treatment completion rate for the main phase [70%] and an assumed participant willingness to participate in the extension [80%] to determine the number of sites required to achieve the target participant number). All participants from the selected sites could enter the extension, provided they met the extension phase eligibility criteria. To be eligible for the extension, participants were required to have completed treatment with semaglutide 2.4 mg or placebo at week 68 and to provide informed consent for the extension. Exclusion criteria included pregnancy or intention of becoming pregnant during the extension and any factor that could have jeopardized compliance (as judged by the investigator).

The STEP 1 trial (including the extension) complied with the International Conference on Harmonisation Good Clinical Practice Guideline and the Declaration of Helsinki. The protocol and amendments were approved by the relevant institutional review board or independent ethics committee at each trial site.

### 2.2 Procedures

The first two visits of the off‐treatment observational extension phase coincided with the week 68 and week 75 visits in the main phase, with three further visits conducted at weeks 80, 104 and 120 (Figure S1). Body measurements, vital sign recording and laboratory assessments (HbA1c, lipid levels and C‐reactive protein [CRP]) were undertaken at each visit. No systematic collection of adverse events was performed in the extension. Investigators and participants remained blinded to the original treatment allocation until extension completion.

No treatments were actively administered or prohibited during the extension. Concomitant treatments or events that might impact weight (obesity pharmacotherapy or bariatric surgery; medications for hypertension, diabetes or reducing lipids; pregnancy) were recorded during the extension phase. Participation in lifestyle interventions that might impact weight were not recorded.

Participants unable to attend site visits because of the COVID‐19 pandemic were offered telephone visits as an alternative. Analyses were limited to data recorded during onsite visits; data obtained from telephone visits were self‐reported and therefore not evaluated.

### 2.3 Outcomes

The extension addressed two exploratory objectives: (1) to examine the change in body weight and cardiometabolic risk factors in participants who completed treatment in the main phase and were followed during the off‐treatment period; and (2) to evaluate the consistency of the 68‐week treatment effect of semaglutide (relative to placebo) in participants in the main phase and extension phase.

Endpoints included changes (from week 68 to week 120 for the first objective; from week 0 to week 68 for the second objective) in body weight (% and kg), BMI, systolic and diastolic blood pressure (SBP and DBP), CRP, HbA1c and lipids, including triglycerides, total cholesterol, high‐density lipoprotein (HDL) cholesterol, low‐density lipoprotein (LDL) cholesterol, very‐low‐density lipoprotein (VLDL) cholesterol and free fatty acids (the latter for the second exploratory objective only, because of different fasting conditions in the main and extension phases). HbA1c was also used to determine glycaemic category (normoglycaemia, prediabetes or diabetes, as per American Diabetes Association HbA1c criteria^21^) and changes in the proportion of participants in each category were assessed. Post hoc analyses explored changes in body weight from baseline to week 120 and in a variety of participant subgroups (from baseline to week 68 and week 120, and from week 68 to week 120), and the proportion of participants with 5% or higher weight loss from baseline at week 120.

### 2.4 Statistical analysis

No power calculations were performed to determine the sample size. All extension phase analyses were exploratory and performed in the extension analysis set (ExAS), which included all participants eligible for the extension who attended at least one visit on week 75, 80, 104 or 120.

Endpoints addressing the first exploratory objective were analysed descriptively using observed data from the in‐trial period (the time from randomization to last contact with a trial site) for the ExAS. No estimands were specified.

Endpoints addressing the second exploratory objective were analysed descriptively and statistically using data from the in‐trial period for the ExAS. Results for the ExAS were compared with results previously reported for the full analysis set (FAS),^16^ which included all participants randomized in the main phase according to the intention‐to‐treat principle. For the statistical analyses reported herein, the treatment policy estimand was used to quantify the 68‐week treatment effect of semaglutide (relative to placebo) in all participants, irrespective of treatment adherence or initiation of other obesity therapies. Week 68 responses were examined using an analysis of covariance model with randomized treatment as factor and baseline endpoint value as covariate. Missing observations were multiply (x 1000) imputed from participants within the same randomized treatment arm with available measurements at week 68. Results from statistical analyses were accompanied by two‐sided 95% confidence intervals (CIs).

Percentage changes in body weight and selected cardiometabolic risk factors were calculated relative to the baseline value of the variable.

## 3 RESULTS

### 3.1 Study participants

From September 2019 to April 2020, 336 participants from the main phase were screened for the extension and 333 entered the extension, including 232 participants who received semaglutide during the main phase (referred to as the “semaglutide arm” hereafter) and 101 who received placebo during the main phase (“placebo arm” hereafter). In total, 327 participants (98.2%) were included in the ExAS (Figure S2). The majority of participants completed the extension (93.7%; n = 312/333). Data from the final week 120 visit were available from site visits for 290 participants (semaglutide arm: n = 197; placebo arm: n = 93). An additional 22 participants completed the end‐of‐trial visit via telephone or email contact.

When assessed according to anatomical therapeutic chemical classification system codes, obesity pharmacotherapies were initiated or ongoing in the extension in two (0.9%) and three (3.0%) participants in the semaglutide and placebo arms, respectively. As medications may have been used off‐label for weight management, obesity pharmacotherapy use was also assessed using investigator‐reported medication classification. Using this definition, there were 21 (9.2%) obesity pharmacotherapy users in the semaglutide arm and six (6.1%) in the placebo arm during the extension, including GLP‐1 receptor agonist use in 14 (6.1%) and four (4.0%) participants in the semaglutide and placebo arms, respectively (including semaglutide up to 1.0 mg and liraglutide up to 3.0 mg). Mean duration of investigator‐reported obesity pharmacotherapy usage in the extension phase was 172.1 and 264.7 days in the semaglutide and placebo arms, respectively (range: 8‐364 days). No participants became pregnant or had bariatric surgery during the extension.

Demographic and clinical characteristics at baseline were generally well balanced across the two arms in the ExAS (Table 1). Most participants were female (67.0%) and White (75.8%), with a mean age of 49.0 years, weight of 105.5 kg and BMI of 37.6 kg/m^2^. Prediabetes was present in 59.6% of participants. The ExAS and FAS had similar demographics and clinical characteristics at baseline.

**TABLE 1:** Demographic and clinical characteristics at baseline

Characteristic	ExAS		FAS	
	Semaglutide arm (N = 228)	Placebo arm (N = 99)	Semaglutide arm (N = 1306)	Placebo arm (N = 655)
Age (y), mean ± SD	48 ± 12	50 ± 11	46 ± 13	47 ± 12
Female sex, n (%)	152 (66.7)	67 (67.7)	955 (73.1)	498 (76.0)
Race or ethnic group, n (%)^a^				
White	174 (76.3)	74 (74.7)	973 (74.5)	499 (76.2)
Asian	43 (18.9)	23 (23.2)	181 (13.9)	80 (12.2)
Black or African American	9 (3.9)	1 (1.0)	72 (5.5)	39 (6.0)
Other	2 (0.9)	1 (1.0)	80 (6.1)	37 (5.6)
Hispanic or Latino ethnicity, n (%)^a^	4 (1.8)	1 (1.0)	150 (11.5)	86 (13.1)
Body weight (kg), mean ± SD	105.6 ± 21.8	105.4 ± 25.6	105.4 ± 22.1	105.2 ± 21.5
Body mass index (kg/m^2^)				
Mean ± SD	37.6 ± 7.0	37.7 ± 8.0	37.8 ± 6.7	38.0 ± 6.5
Distribution, n (%)				
<30	18 (7.9)	12 (12.1)	81 (6.2)	36 (5.5)
≥30 ‐ <35	81 (35.5)	30 (30.3)	436 (33.4)	207 (31.6)
≥35 ‐ <40	64 (28.1)	31 (31.3)	406 (31.1)	208 (31.8)
≥40	65 (28.5)	26 (26.3)	383 (29.3)	204 (31.1)
HbA1c (%), mean ± SD	5.7 ± 0.3	5.7 ± 0.3	5.7 ± 0.3	5.7 ± 0.3
Prediabetes, n (%)^b^	142 (62.3)	53 (53.5)	593 (45.4)	263 (40.2)
Blood pressure (mmHg), mean ± SD				
Systolic	129 ± 14	130 ± 15	126 ± 14	127 ± 14
Diastolic	81 ± 10	80 ± 10	80 ± 10	80 ± 10
Pulse (beats/min), mean ± SD	71 ± 10	70 ± 11	72 ± 10	72 ± 10
Lipid levels (mg/dl), geo mean (CV%)^c^				
Total cholesterol	193.4 (18.4)	194.8 (19.4)	189.6 (20.5)	192.1 (19.4)
HDL cholesterol	49.3 (23.9)	48.9 (26.8)	49.4 (25.6)	49.5 (25.0)
LDL cholesterol	113.4 (29.4)	113.7 (27.2)	110.3 (31.6)	112.5 (29.8)
VLDL cholesterol	25.5 (44.7)	25.9 (54.1)	24.5 (45.8)	24.9 (46.5)
Free fatty acids	12.7 (59.6)	13.7 (52.6)	12.3 (57.9)	12.7 (53.8)
Triglycerides	131.1 (46.5)	132.7 (53.7)	126.2 (47.4)	127.9 (49.0)
Co‐existing conditions, n (%)^d^				
Dyslipidaemia	95 (41.7)	40 (40.4)	499 (38.2)	226 (34.5)
Hypertension	92 (40.4)	38 (38.4)	472 (36.1)	234 (35.7)
Knee osteoarthritis	25 (11.0)	19 (19.2)	173 (13.2)	102 (15.6)
Obstructive sleep apnoea	19 (8.3)	10 (10.1)	159 (12.2)	71 (10.8)
Asthma or COPD	37 (16.2)	10 (10.1)	147 (11.3)	80 (12.2)
Non‐alcoholic fatty liver disease	21 (9.2)	16 (16.2)	101 (7.7)	62 (9.5)
Polycystic ovarian syndrome^e^	19/152 (12.5)	7/67 (10.4)	62/955 (6.5)	34/498 (6.8)
Coronary artery disease	6 (2.6)	2 (2.0)	32 (2.5)	17 (2.6)

*Note*. Data presented for the FAS are from *N Engl J Med*, Wilding JPH, Batterham RL, Calanna S, et al., Once‐weekly semaglutide in adults with overweight or obesity, 384:989‐1002. Copyright © 2021 Massachusetts Medical Society. Reprinted with permission from Massachusetts Medical Society.

Abbreviations: COPD, chronic obstructive pulmonary disease; CV, coefficient of variation; ExAS, extension analysis set; FAS, full analysis set; geo, geometric; HDL, high‐density lipoprotein; LDL, low‐density lipoprotein; n, number of participants; SD, standard deviation; VLDL, very‐low‐density lipoprotein.

^a^ Race and ethnic group were reported by the investigator. The category of “other” includes Native American, Hawaiian or other Pacific Islander, any other ethnic group and “not applicable”, the last of which is the way race or ethnic group was recorded in France.

^b^ In the ExAS, the presence of prediabetes was determined from HbA1c assessments, as per American Diabetes Association HbA1c criteria^21^; prediabetes was defined by HbA1c 5.7%‐6.4% (39‐47 mmol/mol). In the FAS, the presence of prediabetes was determined by investigators on the basis of available information (e.g. medical records, concomitant medication and blood glucose variables) and in accordance with American Diabetes Association HbA1c criteria.^21^

^c^ In the ExAS, baseline lipid levels were reported for 222‐227 participants per variable in the semaglutide group and 97‐98 participants per variable in the placebo group. In the FAS, baseline lipid levels were reported for 1281‐1301 participants per variable in the semaglutide group and 645‐649 participants per variable in the placebo group.

^d^ Selected co‐existing conditions of interest, based on a history of the reported conditions at screening.

^e^ Data on polycystic ovarian syndrome include only female participants; data presented are number of participants/total number of female participants (%).

### 3.2 Change in body weight and BMI

**FIGURE 1:**
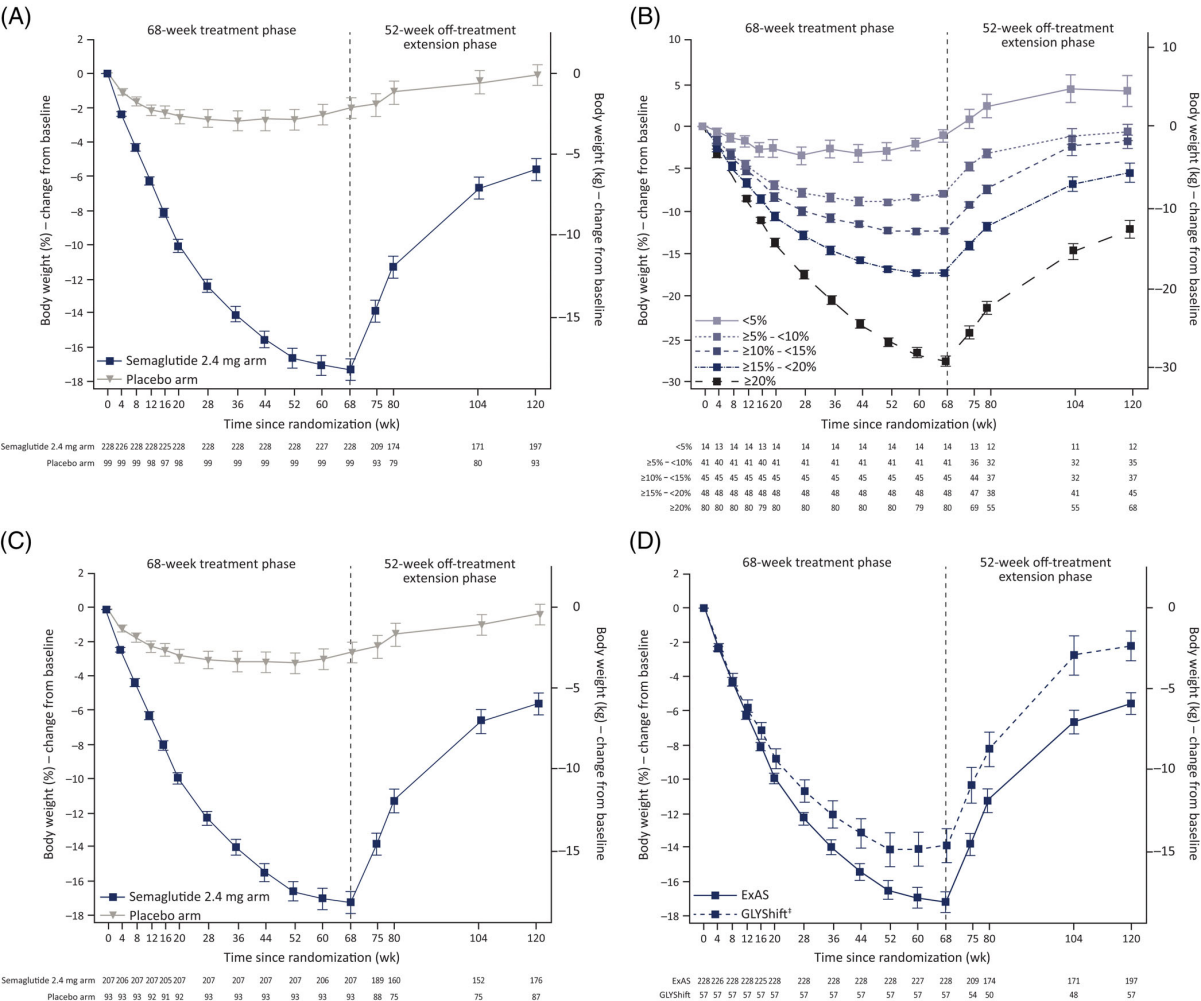
Change from baseline in body weight by week for A, All participants in the ExAS, B, Participants in the semaglutide arm, grouped by categorical weight loss from week 0 to week 68, C, Participants not using obesity pharmacotherapy during the extension^†^, and D, Participants in the semaglutide arm for the full ExAS and the subgroup with prediabetes resolution at week 68 and subsequent reversion by week 120^‡^. ^†^Participants who did not use obesity pharmacotherapies (investigator‐assessed) during the extension phase. ^‡^Participants who shifted from prediabetes at baseline to normoglycaemia at week 68 to prediabetes at week 120. Glycaemic category was determined from HbA1c assessments, as per American Diabetes Association HbA1c criteria.^21^ Normoglycaemia was defined by HbA1c < 5.7% (< 39 mmol/mol); prediabetes was defined by HbA1c 5.7%‐6.4% (39‐47 mmol/mol). Data are observed mean changes from baseline (± standard error) for the ExAS from the in‐trial period. The dashed vertical line at week 68 indicates the end of the main phase and start of the off‐treatment extension phase. Numbers shown in the lower panels are participants contributing to the mean. ExAS, extension analysis set

During the main treatment phase (from baseline [week 0] to week 68), semaglutide reduced body weight more than placebo (Figure 1A and Tables 2 and S1); observed mean weight loss was 17.3% (standard deviation [SD]: 9.3%) with semaglutide versus 2.0% (SD: 6.1%) with placebo (Table S1). After treatment withdrawal, body weight regain was observed in both the semaglutide and placebo arms (Figure 1A and Tables 2 and S1). Participants regained a mean of 11.6 percentage points (SD: 7.7) of body weight in the semaglutide arm versus 1.9 percentage points (SD: 4.8) in the placebo arm (Table S1). The net mean body weight loss over the full duration of the main treatment phase and off‐treatment extension phase (from week 0 to week 120) was 5.6% (SD: 8.9%) in the semaglutide arm versus 0.1% (SD: 5.8%) in the placebo arm (Figure 1A). At week 120, 5% or higher weight loss from baseline was observed in 48.2% of participants (95 of 197) in the semaglutide arm and in 22.6% (21 of 93) in the placebo arm.

**TABLE 2:** Observed body weight, body mass index, cardiovascular risk factors and glucose metabolism in the ExAS at baseline, week 68 and week 120

	Baseline (week 0)				Week 68				Week 120			
	Semaglutide arm		Placebo arm		Semaglutide arm		Placebo arm		Semaglutide arm		Placebo arm	
	N	Mean	N	Mean	N	Mean	N	Mean	N	Mean	N	Mean
Body weight (kg), mean ± SD	228	105.6 ± 21.8	99	105.4 ± 25.6	228	87.5 ± 21.4	99	103.2 ± 25.6	197	99.0 ± 22.5	93	105.5 ± 26.2
Body mass index (kg/m^2^), mean ± SD	228	37.6 ± 7.0	99	37.7 ± 8.0	228	31.2 ± 7.2	99	36.9 ± 8.0	197	35.0 ± 7.1	93	37.6 ± 8.2
Systolic blood pressure (mmHg), mean ± SD	228	129 ± 14	99	130 ± 15	228	121 ± 14	99	128 ± 13	197	131 ± 15	93	132 ± 15
Diastolic blood pressure (mmHg), mean ± SD	228	81 ± 10	99	80 ± 10	228	78 ± 11	99	79 ± 9	197	82 ± 10	93	81 ± 11
HbA1c (%), mean ± SD	228	5.7 ± 0.3	99	5.7 ± 0.3	227	5.2 ± 0.3	98	5.5 ± 0.4	196	5.6 ± 0.3	91	5.7 ± 0.5
Lipid levels (mg/dl), geo mean (CV%)^a^												
Total cholesterol	227	193.4 (18.4)	97	194.8 (19.4)	228	184.6 (20.9)	99	194.9 (19.7)	195	191.4 (19.7)	92	193.4 (20.2)
HDL cholesterol	227	49.3 (23.9)	97	48.9 (26.8)	228	52.8 (23.8)	99	50.1 (26.2)	193	53.1 (26.2)	92	49.4 (25.2)
LDL cholesterol	227	113.4 (29.4)	97	113.7 (27.2)	228	108.2 (32.0)	99	115.0 (32.8)	194	108.5 (30.4)	92	108.8 (33.4)
VLDL cholesterol	227	25.5 (44.7)	97	25.9 (54.1)	228	18.6 (49.1)	99	23.4 (52.4)	194	23.5 (52.7)	92	27.4 (56.4)
Triglycerides	227	131.1 (46.5)	97	132.7 (53.7)	228	95.7 (50.1)	99	119.4 (51.9)	194	122.4 (57.3)	92	140.8 (57.7)
C‐reactive protein (mg/L), geo mean (CV%)	228	2.95 (170.1)	98	3.08 (112.8)	228	1.28 (211.6)	99	2.69 (142.9)	195	1.83 (183.9)	93	2.65 (152.2)

*Note*. Data are for the ExAS from the in‐trial period.

Abbreviations: CV, coefficient of variation; ExAS, extension analysis set; geo, geometric; HDL, high‐density lipoprotein; LDL, low‐density lipoprotein; N, number of participants in the ExAS; SD, standard deviation; VLDL, very‐low‐density lipoprotein.

^a^ Free fatty acids are not reported because of different fasting requirements in the main phase (weeks 0‐68) and the extension phase (weeks 75‐120).

With regard to consistency of the treatment effect between ExAS and FAS, in the ExAS the estimated mean change from week 0 to week 68 was –17.3% with semaglutide versus –2.0% with placebo (estimated treatment difference [ETD]: –15.3%; 95% CI: –17.3%, –13.3%). Respective values for the FAS were –14.9% versus –2.4% (ETD: –12.4%; 95% CI: –13.4%, –11.5%).

Changes in BMI during the main treatment phase and extension phase were consistent with changes in body weight and are reported in Tables 2, S1 and S2.

### 3.3 Change in body weight in participant subgroups

Changes from baseline in body weight at week 120 differed according to the weight lost by week 68. Subgroups with greater weight losses from week 0 to week 68 tended to have numerically greater weight regains from week 68 to week 120, but maintained numerically greater net weight losses from week 0 to week 120 (Figure 1B and Table S3). Participants in the semaglutide arm with weight losses (i.e. < 5%, ≥ 5%‐< 10%, ≥ 10%‐< 15%, ≥ 15%‐< 20% and ≥ 20%) from week 0 to week 68 had net weight changes from week 0 to week 120 of 4.2% (SD: 6.2%), –0.7% (SD: 5.9%), –1.8% (SD: 5.0%), –5.5% (SD: 7.2%) and –12.1% (SD: 8.9%), respectively. In absolute terms, respective changes from week 0 to week 120 for these subgroups were 4.8 (SD: 7.3), –0.9 (SD: 6.7), –2.1 (SD: 5.3), –6.4 (SD: 9.6) and –12.2 (SD: 9.5) kg.

Changes in body weight differed according to participants' baseline characteristics (Table S4). In the semaglutide arm, mean changes from week 0 to week 120 were numerically greater for women versus men; higher versus lower baseline BMI categories; and participants with normoglycaemia versus prediabetes at baseline. Mean changes in body weight from week 0 to week 120 in the semaglutide arm were similar across subgroups defined by baseline age tertiles (Table S4). Changes in body weight from week 0 to week 68 and from week 68 to week 120 for these subgroups are reported in Table S4.

When obesity pharmacotherapy users were excluded, changes in body weight (Figure 1C) were similar to the full ExAS (Figure 1A). Among the subgroup not using obesity pharmacotherapy, mean changes in body weight from week 0 to week 120 were –5.6% (SD: 8.9%) and –0.3% (SD: 5.3%) in the semaglutide and placebo arms, respectively.

### 3.4 Change in cardiometabolic risk factors

**FIGURE 2:**
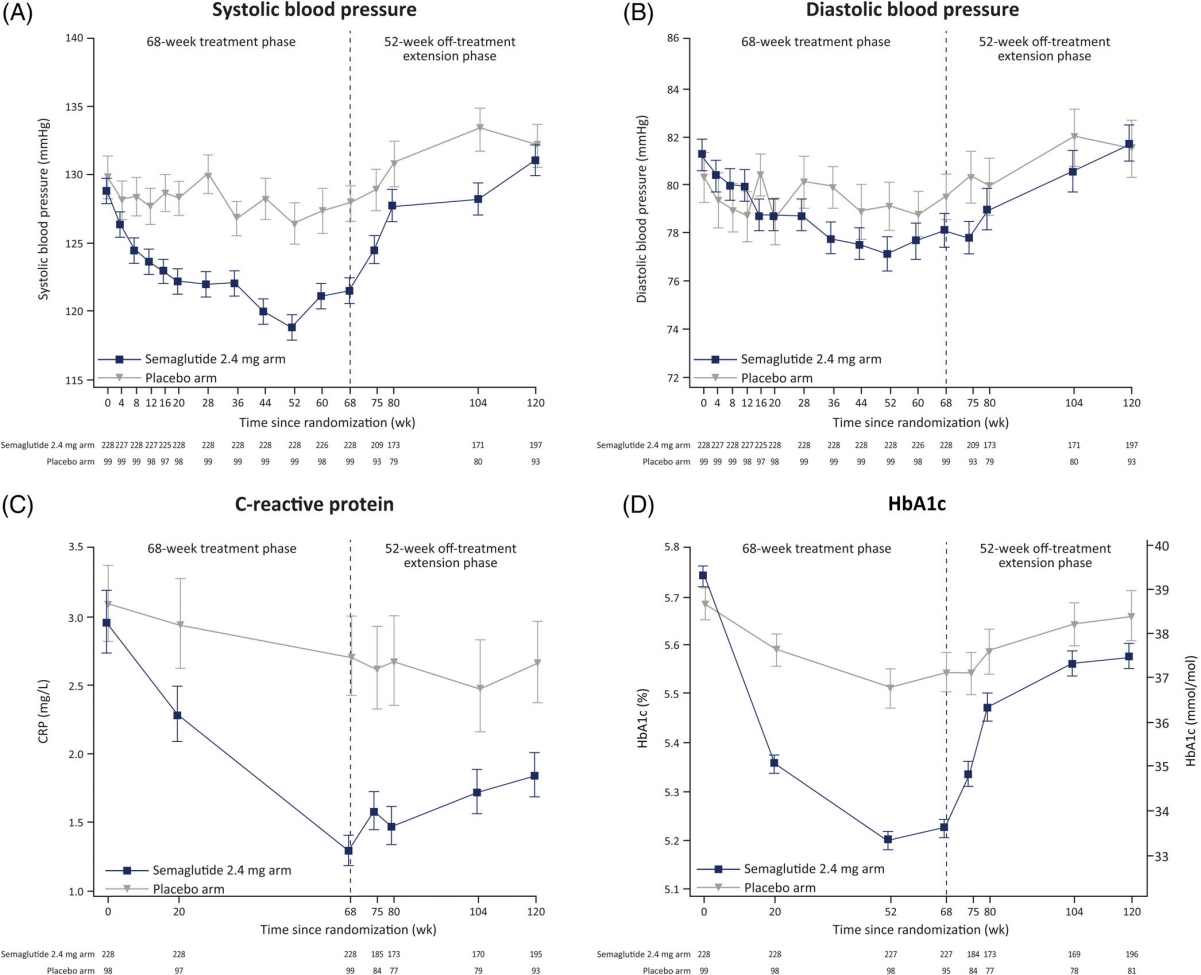
A, Systolic blood pressure, B, Diastolic blood pressure, C, C‐reactive protein, and D, HbA1c by week. Data are observed means (± standard error) for the extension analysis set from the in‐trial period; for C‐reactive protein, standard error was calculated on the logarithmic scale and back‐transformed to the linear scale. The dashed vertical line at week 68 indicates the end of the main phase and start of the off‐treatment extension phase. Numbers shown in the lower panels are participants contributing to the mean. CRP, C‐reactive protein

During treatment, greater improvements in cardiovascular risk factors were observed from week 0 to week 68 with semaglutide than placebo (Tables 2 and S1), which tended to be slightly larger in the ExAS than in the FAS (Table S2). After treatment withdrawal, mean SBP and DBP increased in both treatment arms, reverting to baseline levels by week 120 (Figure 2 and Tables 2 and S1). CRP and lipids increased from week 68 to week 120 in the semaglutide arm, but remained improved relative to the placebo arm for CRP, HDL cholesterol, VLDL cholesterol and triglycerides at week 120 (Figures 2 and S3 and Tables 2 and S1). Improvements were observed in some cardiovascular risk factors from baseline to week 120 (Figures 2 and S3 and Table 2), including for LDL cholesterol and CRP in both semaglutide and placebo arms and for HDL cholesterol, VLDL cholesterol and triglycerides in the semaglutide arm.

During treatment, a greater decrease in HbA1c was observed from week 0 to week 68 with semaglutide than placebo (Tables 2 and S1), which was slightly larger in the ExAS than in the FAS (Table S2). After treatment withdrawal, increases in mean HbA1c were observed in both treatment arms. Although the magnitude of increase from week 68 to week 120 was greater in the semaglutide arm than in the placebo arm (Table S1), the semaglutide arm maintained a small relative improvement versus the placebo arm in HbA1c at week 120 (Figure 2). Compared with baseline, at week 120, observed mean HbA1c was reduced in the semaglutide arm and similar in the placebo arm (Figure 2, Table 2).

In the semaglutide group, greater magnitudes of weight loss from week 0 to week 68 tended to be associated with the most favourable changes in cardiometabolic risk factors from baseline at week 120 (Tables S5 and S6).

### 3.5 Change in glycaemic category

**FIGURE 3:**
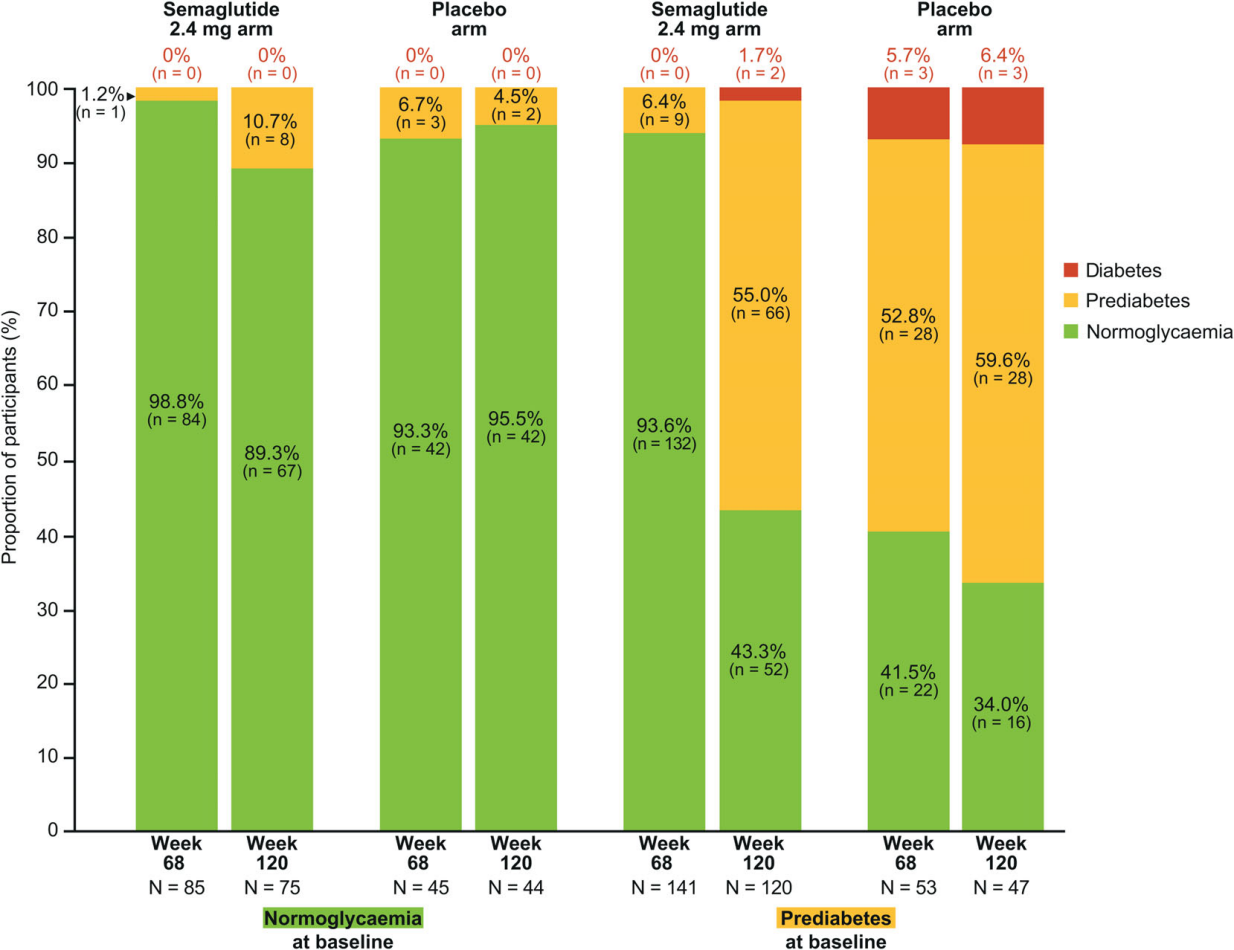
Change in glycaemic category from baseline (week 0) to weeks 68 and 120. Data are observed proportions (%) of participants for the extension analysis set from the in‐trial period. Proportions are based on participants with an observation at the visit. Glycaemic category was determined from HbA1c assessments, as per American Diabetes Association HbA1c criteria.^21^ Normoglycaemia was defined by HbA1c < 5.7% (< 39 mmol/mol); prediabetes was defined by HbA1c 5.7%‐6.4% (39‐47 mmol/mol); diabetes was defined by HbA1c ≥ 6.5% (≥ 48 mmol/mol)

Among participants with prediabetes at baseline, numerically more participants reverted to normoglycaemia at week 68 with semaglutide than placebo (93.6% vs. 41.5%; Figure 3). After treatment withdrawal, improvements deteriorated in both arms. Although the deterioration was larger in the semaglutide arm, a relative improvement was maintained versus the placebo arm at week 120, with reversion to normoglycaemia in 43.3% versus 34.0% of participants with baseline prediabetes in the semaglutide and placebo arms, respectively. Changes in body weight in the subgroup of participants who shifted from prediabetes at baseline to normoglycaemia at week 68 and then reverted to prediabetes by week 120 are shown in Figure 1D and Table S4.

Few participants with normoglycaemia at baseline had progression to prediabetes at week 68 or week 120, in either of the semaglutide or placebo arms (Figure 3).

## 4 DISCUSSION

In STEP 1, 68 weeks of treatment with once‐weekly s.c. semaglutide 2.4 mg plus lifestyle intervention provided significant, clinically relevant reductions in body weight versus placebo among adults with overweight/obesity.^16^ After withdrawal of semaglutide and structured lifestyle intervention, participants regained a mean of two‐thirds of their prior weight loss in the 1‐year off‐treatment extension phase; weight regain continued until the end of follow‐up (week 120). However, some treatment effects were sustained and weight remained 5.6% below baseline in the semaglutide arm. Almost half of the participants in the semaglutide arm (48.2%) still had clinically meaningful weight loss of 5% or more from baseline at week 120, although this proportion represented a substantial fall from that originally achieved at the end of 68 weeks of treatment (86.4%).^16^ Subgroup analyses suggested that participants in the semaglutide arm with greater weight loss during the 68‐week treatment period tended to have greater regain in body weight after semaglutide withdrawal, but ultimately retained greater weight loss at week 120 versus subgroups who lost less weight during the 68‐week treatment period.

Weight regain has previously been observed following withdrawal of obesity pharmacotherapies, including orlistat and lorcaserin—and also semaglutide, as shown in STEP 4—despite continued lifestyle intervention.^13^, ^14^, ^15^ Taken together, these findings and those of the present study confirm the chronicity of obesity and highlight the importance of maintaining long‐term pharmacological treatment for weight management in people with obesity.

In the STEP 1 extension, weight regain was comparatively rapid following semaglutide withdrawal compared with that seen in other obesity pharmacotherapy withdrawal trials, including after semaglutide withdrawal in STEP 4.^13^, ^14^, ^15^ This may well relate to participants who received semaglutide in STEP 1 having achieved greater weight loss prior to withdrawal than in other trials, and thus having greater potential for regain, driven by physiological and behavioural factors.^22^, ^23^ Accordingly, the steepest trajectory of weight regain after withdrawal was observed in participants who had lost 20% or more of baseline body weight during treatment. Furthermore, the absence of structured lifestyle intervention following semaglutide withdrawal contrasts with the continuation of lifestyle intervention in other withdrawal trials (including in STEP 4)^13^, ^14^, ^15^ and may also have contributed to the trajectory of weight regain.

Mean body weight data suggested a slowing of weight regain towards the end of the extension phase in participants withdrawn from semaglutide. By contrast, the STEP 4 study did not show a clear slowing of weight regain towards the end of its 48‐week observation period following semaglutide withdrawal.^14^ However, interpretation of weight trajectory in STEP 4 is limited by the comparatively small magnitude of weight regain (owing to the shorter treatment duration and smaller initial weight loss in STEP 4 relative to STEP 1, and the continuation of lifestyle intervention).^14^ Whether the slowing in regain in the off‐treatment STEP 1 extension represents a plateauing of mean body weight below baseline levels, or simply a slowing of weight regain as body weight approached baseline, cannot be determined without longer follow‐up.

Subgroup analyses indicated no difference in weight loss based on age, but did suggest lesser mean weight loss in men relative to women in the semaglutide arm, both at the end of active treatment (at week 68) and at the end of the off‐treatment extension phase (at week 120). Despite the small difference between men and women, weight losses during treatment were still substantial and clinically meaningful in both sexes. Subgroup analyses based on baseline BMI categories showed inconsistent trends in the semaglutide arm: weight loss during treatment (week 0 to week 68) showed no clear trend across increasing BMI categories, but over week 0 to week 120 showed a trend for greater weight loss in the subgroups of patients with higher versus lower baseline BMI categories, in part owing to a lesser weight regain in the 40 kg/m^2^ or higher subgroup than most others. However, the differences in weight loss between baseline BMI subgroups were comparatively small, and a meaningful effect of baseline BMI cannot be concluded based on the present data. Differences in weight loss in the semaglutide arm were also seen based on baseline glycaemic status, with lesser weight loss from baseline at week 68 and week 120 among those with prediabetes at baseline relative to those with normoglycaemia. This finding is consistent with the established difficulty of achieving weight loss among people with type 2 diabetes, and with the previous observation of smaller reductions in body weight with semaglutide 2.4 mg in patients with overweight/obesity and type 2 diabetes in the STEP 2 trial,^24^ relative to those seen during the main treatment period of STEP 1, which excluded patients with type 2 diabetes.^16^

We observed residual benefits in some cardiovascular risk factors at week 120 compared with baseline. Previously, a post hoc analysis of the Look AHEAD trial explored the effects of weight regain on cardiometabolic risk factors among people with overweight/obesity and type 2 diabetes who received intensive lifestyle intervention.^25^ In that analysis, large initial weight losses followed by partial or full regain by year 4 were associated with a sustained benefit on HbA1c at year 4 relative to that seen in participants with smaller or no initial weight loss.^25^ In the STEP 1 extension, the semaglutide arm maintained a small improvement from baseline in HbA1c, and also in other cardiometabolic risk factors (LDL, VLDL and HDL cholesterol, triglycerides and CRP) after 1 year off‐treatment, despite partial weight regain. Although many participants who reverted from prediabetes to normoglycaemia during semaglutide treatment subsequently returned to prediabetes after withdrawal, the semaglutide arm maintained a relative improvement at week 120 compared with the placebo arm. Reversion from normoglycaemia to prediabetes after semaglutide withdrawal may relate to loss of the direct effects of GLP‐1 receptor agonism on glycaemic levels. However, the present study does not allow definitive conclusions regarding mechanisms driving changes in glycaemic status. Overall, although a net beneficial effect on several cardiometabolic variables was maintained 1 year after semaglutide withdrawal, greater benefits were seen during the treatment period, supporting the need for continued treatment. The benefits remaining at 1 year post‐withdrawal appear to be related to the magnitude of initial weight loss at week 68. A longer follow‐up study is needed to determine if these remaining benefits would ultimately be lost or retained.

The strengths of the STEP 1 extension include the pragmatic trial design, with no active intervention and infrequent site contact. This more closely mimics a real‐world setting and may aid understanding of real‐world effects of semaglutide withdrawal. Additional strengths include the multinational setting and the high rates of trial completion. The key limitations of the extension were the relatively small sample size compared with the main STEP 1 trial population and the selection of sites based on those with the highest recruitment in the STEP 1 main phase, which could introduce an element of selection bias. However, participants were from a range of countries representative of those included in the main phase, and comparison of baseline characteristics among the ExAS and FAS indicated that the extension sample was representative. Weight losses with semaglutide versus placebo from week 0 to week 68 were slightly greater in the ExAS than in the FAS, as expected given that the ExAS only included participants who completed 68 weeks of treatment. Another limitation was the potential for obesity pharmacotherapy use during the extension. However, few participants used obesity pharmacotherapy so our findings were not confounded, as shown by the consistency of body weight changes in the overall ExAS and the subgroup of participants not using obesity pharmacotherapy. In addition, all analyses were exploratory and thus no formal tests of statistical significance were performed.

In conclusion, among adults with overweight/obesity, after a substantial reduction in body weight during 68 weeks of treatment with once‐weekly s.c. semaglutide plus lifestyle intervention, subsequent treatment withdrawal led to most of the weight loss being regained within 1 year, and a similar change in some cardiometabolic variables back to baseline, reinforcing the need for continued treatment to maintain weight loss and cardiometabolic benefits.

## AUTHOR CONTRIBUTIONS

JPHW contributed to the conduct of the trial, data collection, analysis and interpretation and manuscript development. RLB contributed to the conduct of the trial, data collection, analysis and interpretation and manuscript development. MD contributed to the data interpretation and manuscript development. LFVG contributed to the conduct of the trial, data collection and interpretation and manuscript development. KKa contributed to the data analysis and interpretation and manuscript development. KKo contributed to the data analysis and interpretation and manuscript development. IL contributed to the data interpretation and manuscript development. BMM contributed to the conduct of the trial, data collection, analysis and interpretation and manuscript development. TKO contributed to the data analysis and interpretation and manuscript development. JR contributed to the conduct of the trial, data collection, analysis and interpretation and manuscript development. TAW contributed to the data interpretation and manuscript development. SW contributed to the conduct of the trial, data collection, analysis and interpretation and manuscript development. KY contributed to the data interpretation and manuscript development. RFK contributed to the conduct of the trial, data collection, analysis and interpretation and manuscript development.

## CONFLICT OF INTEREST

JPHW reports receiving advisory board fees, paid to his institution, from Astellas Pharma, grant support and fees for membership on a data and safety monitoring board, both paid to the University of Liverpool, lecture fees and travel support from AstraZeneca, advisory board fees, paid to his institution, and lecture fees from Boehringer Ingelheim, Napp and Sanofi Pasteur, advisory board fees, paid to his institution, from Eli Lilly, Janssen Global Services, Rhythm and Wilmington Healthcare, lecture fees from Mundipharma, grant support, advisory board fees and fees for serving as an investigator, all paid to the University of Liverpool, and lecture fees from Novo Nordisk and advisory board fees from Takeda Medical Research Foundation. RLB reports research grant support from Novo Nordisk and consultancy with Boehringer Ingelheim, Eli Lilly, Gila Therapeutics Inc, GSK, Novo Nordisk, and Pfizer. MD reports receiving research funding from AstraZeneca, Boehringer Ingelheim, Janssen, Novo Nordisk and Sanofi‐Aventis, and has acted as a consultant, advisory board member and speaker for Boehringer Ingelheim, Eli Lilly, Novo Nordisk and Sanofi‐Aventis, as an advisory board member and speaker for AstraZeneca, as an advisory board member for Gilead Sciences Ltd, Janssen and Lexicon, and as a speaker for Napp Pharmaceuticals and Takeda Pharmaceuticals International Inc. She is co‐funded by the NIHR Leicester Biomedical Research Centre. LFVG reports receiving lecture fees from AstraZeneca and Boehringer Ingelheim and advisory board fees and lecture fees from Merck and Novo Nordisk. KKa reports being employed by and owning stock in Novo Nordisk. KKo and TKO report being employed by Novo Nordisk. IL reports receiving advisory board fees and/or consulting fees from AstraZeneca, Bayer HealthCare Pharmaceuticals, Boehringer Ingelheim, Eli Lilly, Intarcia, Intercept Pharmaceuticals, Janssen Global Services, MannKind, Novo Nordisk, Sanofi, Target Pharma, Valeritas and Zealand Pharma, and grant support, paid to UT Southwestern, from Merck, Mylan Pharmaceuticals, Novo Nordisk, Pfizer and Sanofi. BMM reports receiving educational fees from AstraZeneca, Merck and Orexigen Therapeutics, lecture fees from Janssen Biotech, advisory board fees from Johnson & Johnson Health Care Systems, grant support, paid to Guy's and St. Thomas' Hospital, consulting fees and educational fees from Novo Nordisk and owning stock in Reset Health Clinics. JR reports receiving grant support, advisory board fees and travel support from Applied Therapeutics, Intarcia and Oramed, grant support and consulting fees from AstraZeneca, grant support, advisory board fees, lecture fees and travel support from Boehringer Ingelheim, Novo Nordisk and Sanofi US Services, grant support and advisory board fees from Eli Lilly, grant support from Genentech, GlaxoSmithKline, Janssen Biotech, Lexicon Pharmaceuticals, Novartis, Pfizer and REMD Biotherapeutics and advisory board fees from Zealand Pharma. TAW reports receiving grant support from Novo Nordisk and Epitomee Medical, paid to the University of Pennsylvania, and personal advisory board fees from Novo Nordisk and WW International. SW reports receiving lecture fees from AstraZeneca and Bausch and Lomb and grant support, lecture fees and advisory board fees from Novo Nordisk. KY reports receiving lecture fees from Amgen, Janssen Pharmaceuticals, Kyowa Hakko Kirin, Novartis Pharma and Sanofi, grant support and lecture fees from Astellas Pharma, Daiichi Sankyo, Eli Lilly Japan, Merck Sharp and Dohme, Mitsubishi Tanabe Pharma, Nippon Boehringer Ingelheim, Novo Nordisk, Ono Pharmaceutical, Pfizer, Sumitomo Dainippon Pharma, Taisho Toyama Pharmaceutical and Takeda Pharmaceutical, advisory board fees and lecture fees from AstraZeneca, grant support, lecture fees and advisory board fees from Kowa Company and Novo Nordisk and lecture fees and advisory board fees from Sanofi. RFK reports receiving advisory board fees from Novo Nordisk and WW International.

### PEER REVIEW

The peer review history for this article is available at https://publons.com/publon/10.1111/dom.14725.

## Supporting information


**Data S1.** Plain Language Summary

**Appendix S1.** Supporting Information

## Data Availability

Data will be shared with bona fide researchers who submit a research proposal approved by the independent review board. Individual participant data will be shared in data sets in a de‐identified and anonymized format. Data will be made available after research completion and approval of the product and product use in the European Union and the USA. Information about data access request proposals can be found at novonordisk‐trials.com.
