# Supplementary material for: Weight regain and cardiometabolic effects after withdrawal of semaglutide: The STEP 1 trial extension
Source: Diabetes Obes Metab. 2022 May 19;24(8):1553–64. doi: 10.1111/dom.14725 (PMC9542252; doi:10.1111/dom.14725)
Supplement: Supplementary file 1 — Data S1. Plain Language Summary [file DOM-24-1553-s001.docx]

**Plain Language Summary for article “*Weight regain and cardiometabolic effects after withdrawal of semaglutide: the STEP 1 trial extension*”**

**John PH Wilding,^1^ Rachel L Batterham,^2–4^ Melanie Davies,^5,6^ Luc F Van Gaal,^7^ Kristian Kandler,^8^ Katerina Konakli,^8^ Ildiko Lingvay,^9^ Barbara M McGowan,^10^ Tugce Kalayci Oral,^8^ Julio Rosenstock,^11^ Thomas A Wadden,^12^ Sean Wharton,^13^ Koutaro Yokote,^14^ Robert F Kushner,^15^ for the STEP 1 Study Group**

**What is the context and purpose of this research study?**

Obesity, excess fat accumulation that impairs health, is a chronic disease associated with several long-term health issues including type 2 diabetes, high cholesterol and blood pressure, heart disease and premature death. Weight loss improves obesity-associated health problems. Achieving substantial sustained weight loss through diet and exercise is difficult because metabolic changes lead to weight re-gain. Medications that target the body’s appetite regulation systems can help people achieve and maintain weight loss, but if these are stopped, weight regain occurs.

A previous study, the STEP 1 study, found that in people with overweight or obesity, treatment with a drug called semaglutide led to considerable weight loss compared with “dummy” (placebo) treatment alongside a lifestyle intervention. This current study looked at what happens in those participants when they stop taking weight loss treatment altogether.

**What was done?**

The STEP 1 study had a main phase and an extension phase. In the main phase, 1961 adults with overweight or obesity received treatment with semaglutide or placebo, as well as counselling about diet and physical activity, for 68 weeks. At the end of the main phase, a total of 327 participants were entered into this extension study, at which point all weight loss treatments were discontinued. These participants were followed for an additional 52 weeks (meaning they were followed for 120 weeks in total during both the main and extension phases).

This extension study looked at: 1) changes in body weight over time, and 2) changes in measures such as blood pressure, lipid levels, blood sugar levels and markers indicating inflammation, all of which indicate a risk of heart disease and metabolic diseases. Changes were measured over a year, starting the day that the participants stopped taking all treatments.

**What were the main results?**

In the 327 participants who took part in the extension, those who received semaglutide during the main phase had lost 17.3% of their body weight while those who had placebo lost 2.0%. After stopping medication and counselling, weight regain occurred in both groups (by 11.6% of their original weight in those who had received semaglutide, and by nearly 1.9% in those who had placebo).This means that by the end of week 120, those originally treated with semaglutide were below their starting weight by 5.6%, whereas those who received placebo essentially returned to their starting weight (0.1% below). Weight regain seen in both groups is consistent with previous observations of weight regain when weight management interventions are discontinued.

Improvements seen in the risk factors for heart disease and metabolic disease during the main phase reverted towards baseline during the extension phase. However, some of the measures (some lipids, inflammation substances, blood sugar levels) were still improved by the end of the extension in those who had originally received semaglutide.

**What is the originality and relevance of this study?**

This is the first study to evaluate the effects on weight and other related metabolic measures after discontinuation of a successful weight loss medication, semaglutide, and builds on the results of previous studies showing that when obesity treatments are stopped, weight regain occurs. Taken together, these findings indicate that obesity is a chronic disease, and reinforce the importance of continued, long-term treatment with effective medications to maintain weight loss and improvements in other measures.

Author affiliations

^1^Department of Cardiovascular and Metabolic Medicine, Institute of Life Course and Medical Sciences, University of Liverpool, Liverpool, UK; ^2^University College London Centre for Obesity Research, Division of Medicine, University College London, London, UK; ^3^National Institute of Health Research, UCLH Biomedical Research Centre, London, UK; ^4^Centre for Weight Management and Metabolic Surgery, University College London Hospital, London, UK; ^5^Diabetes Research Centre, University of Leicester, Leicester, UK; ^6^NIHR Leicester Biomedical Research Centre, Leicester, UK; ^7^Department of Endocrinology, Diabetology and Metabolism, Antwerp University Hospital, University of Antwerp, Antwerp, Belgium; ^8^Novo Nordisk A/S, Søborg, Denmark; ^9^Departments of Internal Medicine/Endocrinology and Department of Population and Data Sciences, University of Texas Southwestern Medical Center, Dallas, Texas, USA; ^10^Department of Diabetes and Endocrinology, Guy's and St Thomas' NHS Foundation Trust, London, UK; ^11^Dallas Diabetes Research Center at Medical City, Dallas, Texas, USA; ^12^Department of Psychiatry, Perelman School of Medicine, University of Pennsylvania, Philadelphia, Pennsylvania, USA; ^13^York University, McMaster University and Wharton Weight Management Clinic, Toronto, Ontario, Canada; ^14^Department of Endocrinology, Hematology and Gerontology, Graduate School of Medicine, Chiba University and Department of Diabetes, Metabolism and Endocrinology, Chiba University Hospital, Chiba, Japan; ^15^Division of Endocrinology, Feinberg School of Medicine, Northwestern University, Chicago, Illinois, USA.
