# Supplementary material for: Weight regain and cardiometabolic effects after withdrawal of semaglutide: The STEP 1 trial extension
Source: Diabetes Obes Metab. 2022 May 19;24(8):1553–64. doi: 10.1111/dom.14725 (PMC9542252; doi:10.1111/dom.14725)
Supplement: Supplementary file 2 — Appendix S1. Supporting Information [file DOM-24-1553-s002.docx]

**Supplementary appendix**

**Contents**

[Supplementary tables 2](#_Toc97197811)

[Supplementary figures 17](#_Toc97197812)

[Supplementary references 22](#_Toc97197813)

# Supplementary tables

## Table S1. Observed changes in body weight, body mass index, cardiovascular risk factors and glucose metabolism in the ExAS

|  | **On-treatment period**  **(baseline to week 68)** | | | | **Off-treatment period  (week 68 to 120)** | | | |
| --- | --- | --- | --- | --- | --- | --- | --- | --- |
|  | **Semaglutide arm** | | **Placebo arm** | | **Semaglutide arm** | | **Placebo arm** | |
|  | **N** | **Change** | **N** | **Change** | **N** | **Change** | **N** | **Change** |
| Mean±SD change from baseline to week 68 and change from week 68 to week 120 |  |  |  |  |  |  |  |  |
| Body weight (%) | 228 | –17.3±9.3 | 99 | –2.0±6.1 | 197 | 11.6±7.7^†^ | 93 | 1.9±4.8^†^ |
|  |  |  |  |  |  | 14.8±10.7^‡^ |  | 2.1±4.9^‡^ |
| Body weight (kg) | 228 | –18.1±10.1 | 99 | –2.2±6.6 | 197 | 12.0±8.4 | 93 | 2.0±4.8 |
| Body mass index (kg/m^2^) | 228 | –6.4±3.6 | 99 | –0.8±2.3 | 197 | 4.3±2.9 | 93 | 0.7±1.7 |
| Systolic blood pressure (mmHg) | 228 | –7±13 | 99 | –2±13 | 197 | 9±14 | 93 | 4±15 |
| Diastolic blood pressure (mmHg) | 228 | –3±9 | 99 | –1±9 | 197 | 4±10 | 93 | 2±10 |
| HbA_1c_ (%) | 227 | –0.5±0.3 | 98 | –0.2±0.2 | 195 | 0.4±0.3 | 90 | 0.1±0.3 |
| Geometric mean (CV%) ratio to baseline at week 68 and ratio to week 68 at week 120 |  |  |  |  |  |  |  |  |
| Total cholesterol | 227 | 0.95 (14.0) | 97 | 1.01 (17.1) | 195 | 1.04 (13.2) | 92 | 0.99 (15.9) |
| HDL cholesterol | 227 | 1.07 (15.6) | 97 | 1.02 (16.0) | 193 | 1.00 (16.0) | 92 | 0.99 (15.7) |
| LDL cholesterol | 227 | 0.95 (20.8) | 97 | 1.02 (32.4) | 194 | 1.01 (20.5) | 92 | 0.95 (25.6) |
| VLDL cholesterol | 227 | 0.73 (39.0) | 97 | 0.90 (36.9) | 194 | 1.27 (41.1) | 92 | 1.16 (37.4) |
| Triglycerides | 227 | 0.73 (40.1) | 97 | 0.90 (37.1) | 194 | 1.29 (42.5) | 92 | 1.17 (38.0) |
| Free fatty acids | 217 | 0.76 (88.1) | 96 | 0.86 (65.0) | N/A^§^ | | N/A^§^ | |
| C-reactive protein | 228 | 0.43 (127.6) | 98 | 0.88 (88.7) | 195 | 1.50 (124.1) | 93 | 1.02 (122.1) |

^†^Changes expressed as percentage change relative to baseline body weight.
^‡^Changes expressed as percentage change relative to week 68 body weight.

^§^Changes in free fatty acids from week 68 to week 120 were not assessed as measurements at weeks 68 and 120 were non-comparable due to differences in fasting requirements in the main phase versus the extension phase.
Data are for the ExAS from the in-trial period.
CV, coefficient of variation; ExAS, extension analysis set; HbA_1c_, glycated haemoglobin; HDL, high-density lipoprotein; LDL, low-density lipoprotein; n, number of participants in the ExAS; SD, standard deviation; VLDL, very-low-density lipoprotein.

## Table S2. Treatment effect in the ExAS versus the FAS for body weight, body mass index, cardiovascular risk factors and glucose metabolism^†^

| **Characteristic** | **ExAS  (N = 327)** | **FAS**^‡^ **(N = 1961)** |
| --- | --- | --- |
| Change from baseline to week 68, ETD (95% CI) |  |  |
| Body weight (%) | –15.26  (–17.27 to –13.26) | –12.44 (–13.37 to –11.51) |
| Body weight (kg) | –15.88  (–18.01 to –13.76) | –12.71  (–13.68 to –11.74) |
| Body mass index (kg/m^2^) | –5.67  (–6.42 to –4.91) | –4.61  (–4.96 to –4.27) |
| Systolic blood pressure (mmHg) | –5.84 (–8.58 to –3.09) | –5.10 (–6.34 to –3.87) |
| Diastolic blood pressure (mmHg) | –1.93 (–3.88 to 0.02) | –2.41 (–3.25 to –1.57) |
| HbA_1c_ (%) | –0.35 (–0.40 to –0.29) | –0.29 (–0.32 to –0.26) |
| Ratio to baseline at week 68, ETR (95% CI) |  |  |
| Total cholesterol | 0.95 (0.92 to 0.99) | 0.97 (0.95 to 0.98) |
| HDL cholesterol | 1.05 (1.01 to 1.09) | 1.04 (1.02 to 1.05) |
| LDL cholesterol | 0.94 (0.89 to 1.00) | 0.96 (0.94 to 0.98) |
| VLDL cholesterol | 0.81 (0.74 to 0.88) | 0.84 (0.81 to 0.87) |
| Triglycerides | 0.81 (0.74 to 0.88) | 0.84 (0.81 to 0.87) |
| Free fatty acids | 0.84 (0.73 to 0.97) | 0.89 (0.83 to 0.94) |
| C-reactive protein | 0.49 (0.40 to 0.61) | 0.56 (0.51 to 0.61) |

^†^The treatment policy estimand was used to quantify the 68-week treatment effect of semaglutide (relative to placebo), irrespective of treatment adherence or initiation of other obesity pharmacotherapies. Week 68 responses were analysed using an analysis of covariance model, with randomized treatment as factor and baseline endpoint value as covariate, and a multiple imputation approach for missing data.
^‡^Data for the FAS were previously presented by Wilding JPH, Batterham RL, Calanna S, Davies M, Van Gaal LF, Lingvay I, et al in ‘Once-weekly semaglutide in adults with overweight or obesity’ (*N Engl J Med* 2021;384:989).
Data are from the in-trial period.
CI, confidence interval; ETD, estimated treatment difference; ETR, estimated treatment ratio; ExAS, extension analysis set; FAS, full analysis set; HbA_1c_, glycated haemoglobin; HDL, high-density lipoprotein; LDL, low-density lipoprotein; n, number of participants in each analysis set; VLDL, very-low-density lipoprotein.

## Table S3. Changes in body weight (%) in subgroups by body weight loss from baseline to week 68

| **Participant subgroups by body weight loss from baseline to week 68** | **Change from  baseline to week 120** | | | | **Change from  baseline to week 68** | | | | **Change from  week 68 to week 120** | | | |
| --- | --- | --- | --- | --- | --- | --- | --- | --- | --- | --- | --- | --- |
|  | **Semaglutide  arm** | | **Placebo arm** | | **Semaglutide  arm** | | **Placebo arm** | | **Semaglutide  arm** | | **Placebo  arm** | |
|  | **N** | **Change,  %±SD** | **N** | **Change,  %±SD** | **N** | **Change,  %±SD** | **N** | **Change, %±SD** | **N** | **Change, %-points**^†^**±SD** | **N** | **Change, %-points**^†^**±SD** |
| <5% subgroup | 12 | 4.2±6.2 | 69 | 1.5±5.5 | 14 | –1.1±2.8 | 73 | 0.8±3.8 | 12 | 4.8±6.7 | 69 | 0.8±4.6 |
| ≥5 – <10% subgroup | 35 | –0.7±5.9 | 16 | –3.2±2.9 | 41 | –8.0±1.4 | 17 | –7.5±1.5 | 35 | 7.3±6.0 | 16 | 4.4±3.1 |
| ≥10 – <15% subgroup | 37 | –1.8±5.0 | 5 | –7.8±5.0 | 45 | –12.4±1.4 | 5 | –11.9±1.3 | 37 | 10.7±5.1 | 5 | 4.1±4.2 |
| ≥15 – <20% subgroup | 45 | –5.5±7.2 | 2 | –7.1±2.0 | 48 | –17.3±1.6 | 3 | –17.1±1.3 | 45 | 11.9±7.1 | 2 | 9.8±0.2 |
| ≥20% subgroup | 68 | –12.1±8.9 | 1 | –9.9±NA | 80 | –27.6±5.5 | 1 | –20.7±NA | 68 | 15.4±8.1 | 1 | 10.8±NA |

^†^Changes expressed as percentage point changes relative to baseline body weight.
Data are observed means ± SD for the extension analysis set from the in-trial period.
NA, not applicable; SD, standard deviation.

## Table S4. Changes in body weight (%) in participant subgroups

|  | **Change from  baseline to week 120** | | | | **Change from  baseline to week 68** | | | | **Change from  week 68 to week 120** | | | |
| --- | --- | --- | --- | --- | --- | --- | --- | --- | --- | --- | --- | --- |
|  | **Semaglutide  arm** | | **Placebo arm** | | **Semaglutide  arm** | | **Placebo arm** | | **Semaglutide  arm** | | **Placebo  arm** | |
| **Subgroup** | **N** | **Change,  %±SD** | **N** | **Change,  %±SD** | **N** | **Change,  %±SD** | **N** | **Change, %±SD** | **N** | **Change, %-points**^†^**±SD** | **N** | **Change, %-points**^†^**±SD** |
| Sex |  |  |  |  |  |  |  |  |  |  |  |  |
| Male | 71 | –4.5±7.6 | 32 | –0.6±5.8 | 76 | –14.5±8.0 | 32 | –2.3±5.5 | 71 | 9.8±6.0 | 32 | 1.7±4.0 |
| Female | 126 | –6.2±9.5 | 61 | 0.1±5.8 | 152 | –18.7±9.6 | 67 | –1.9±6.4 | 126 | 12.7±8.3 | 61 | 2.0±5.1 |
| Age (years) |  |  |  |  |  |  |  |  |  |  |  |  |
| <45 | 74 | –5.5±10.6 | 25 | 1.4±4.8 | 86 | –19.1±10.4 | 27 | –1.6±5.9 | 74 | 13.4±9.6 | 25 | 2.4±3.8 |
| 45 to 54 | 62 | –5.5±8.1 | 33 | –0.2±5.6 | 72 | –17.4±8.7 | 35 | –2.0±6.5 | 62 | 12.0±6.6 | 33 | 2.2±5.4 |
| >54 | 61 | –5.9±7.4 | 35 | –1.1±6.5 | 70 | –15.1±8.1 | 37 | –2.4±6.1 | 61 | 9.1±4.9 | 35 | 1.3±4.8 |
| Baseline body mass index (kg/m^2^) |  |  |  |  |  |  |  |  |  |  |  |  |
| <30 | 16 | –4.5±7.3 | 12 | –0.7±5.8 | 18 | –14.6±9.6 | 12 | –2.2±5.8 | 16 | 9.7±6.7 | 12 | 1.5±7.5 |
| ≥30 to <35 | 76 | –4.8±8.9 | 26 | –0.2±5.7 | 81 | –17.6±9.1 | 30 | –2.0±6.1 | 76 | 12.5±7.2 | 26 | 1.9±4.0 |
| ≥35 to <40 | 53 | –5.6±8.2 | 31 | –0.2±6.0 | 64 | –18.8±9.6 | 31 | –1.8±6.8 | 53 | 12.8±8.1 | 31 | 1.6±5.0 |
| ≥40 | 52 | –7.2±9.9 | 24 | 0.4±5.9 | 65 | –16.2±9.0 | 26 | –2.3±5.8 | 52 | 9.7±7.7 | 24 | 2.5±3.6 |
| Baseline glycaemic category^‡^ |  |  |  |  |  |  |  |  |  |  |  |  |
| Normoglycaemia | 73 | –6.4±9.5 | 44 | 1.2±5.9 | 83 | –19.7±9.5 | 45 | –1.5±5.9 | 73 | 13.7±8.6 | 44 | 2.4±4.3 |
| Prediabetes | 119 | –5.1±8.4 | 47 | –1.4±5.6 | 140 | –15.7±8.8 | 52 | –2.6±6.4 | 119 | 10.2±6.8 | 47 | 1.5±5.2 |
| Shift in glycaemic category^‡^ |  |  |  |  |  |  |  |  |  |  |  |  |
| Prediabetes at baseline to normoglycaemia at week 68 to prediabetes at week 120 | 57 | –2.2±6.5 | 7 | –1.4±5.4 | 57 | –14.0±7.5 | 7 | –4.2±5.6 | 57 | 11.7±6.2 | 7 | 2.7±3.4 |
| Total extension analysis set | 197 | –5.6±8.9 | 93 | –0.1±5.8 | 228 | –17.3±9.3 | 99 | –2.0±6.1 | 197 | 11.6±7.7 | 93 | 1.9±4.8 |

^†^Changes expressed as percentage point changes relative to baseline body weight.
^‡^Glycaemic category was determined from HbA_1c_ assessments, per American Diabetes Association HbA_1c_ criteria.^1^ Normoglycaemia was defined by HbA_1c_ <5.7% (39 mmol/mol); prediabetes was defined by HbA_1c_ 5.7–6.4% (39–47 mmol/mol).
Data are observed means ± SD for the extension analysis set from the in-trial period.
HbA_1c_, glycated haemoglobin; SD, standard deviation.

## Table S5. Cardiometabolic risk factors at baseline, week 68 and week 120, by body weight loss from baseline to week 68 subgroups

|  | **Baseline (week 0)** | | | | **Week 68** | | | | **Week 120** | | | |
| --- | --- | --- | --- | --- | --- | --- | --- | --- | --- | --- | --- | --- |
|  | **Semaglutide arm** | | **Placebo  arm** | | **Semaglutide arm** | | **Placebo  arm** | | **Semaglutide arm** | | **Placebo  arm** | |
|  | **N** | **Mean^†^** | **N** | **Mean^†^** | **N** | **Mean^†^** | **N** | **Mean^†^** | **N** | **Mean^†^** | **N** | **Mean^†^** |
| Systolic blood pressure (mmHg) |  |  |  |  |  |  |  |  |  |  |  |  |
| Weight loss subgroup^‡^: <5% | 14 | 127±12 | 73 | 131±15 | 14 | 132±13 | 73 | 129±13 | 12 | 140±13 | 69 | 133±16 |
| Weight loss subgroup^‡^: ≥5 – <10% | 41 | 131±16 | 17 | 130±16 | 41 | 124±14 | 17 | 126±15 | 35 | 132±19 | 16 | 132±15 |
| Weight loss subgroup^‡^: ≥10 – <15% | 45 | 129±13 | 5 | 124±10 | 45 | 121±13 | 5 | 128±12 | 37 | 134±13 | 5 | 122±12 |
| Weight loss subgroup^‡^: ≥15 – <20% | 48 | 130±12 | 3 | 122±28 | 48 | 123±13 | 3 | 114±12 | 45 | 131±13 | 2 | 144±16 |
| Weight loss subgroup^‡^: ≥20% | 80 | 127±15 | 1 | 128±NA | 80 | 118±15 | 1 | 129±NA | 68 | 128±15 | 1 | 129±NA |
| Diastolic blood pressure (mmHg) |  |  |  |  |  |  |  |  |  |  |  |  |
| Weight loss subgroup^‡^: <5% | 14 | 81±9 | 73 | 81±11 | 14 | 86±10 | 73 | 80±10 | 12 | 86±13 | 69 | 81±12 |
| Weight loss subgroup^‡^: ≥5 – <10% | 41 | 81±10 | 17 | 79±7 | 41 | 79±10 | 17 | 80±10 | 35 | 81±10 | 16 | 83±11 |
| Weight loss subgroup^‡^: ≥10 – <15% | 45 | 80±12 | 5 | 82±3 | 45 | 77±12 | 5 | 82±2 | 37 | 82±11 | 5 | 79±5 |
| Weight loss subgroup^‡^: ≥15 – <20% | 48 | 84±9 | 3 | 75±15 | 48 | 79±9 | 3 | 70±8 | 45 | 83±11 | 2 | 78±7 |
| Weight loss subgroup^‡^: ≥20% | 80 | 80±11 | 1 | 84±NA | 80 | 76±11 | 1 | 83±NA | 68 | 80±10 | 1 | 81±NA |
| HbA_1c_ (%) |  |  |  |  |  |  |  |  |  |  |  |  |
| Weight loss subgroup^‡^: <5% | 14 | 5.9±0.2 | 71 | 5.7±0.3 | 14 | 5.5±0.1 | 72 | 5.6±0.4 | 12 | 6.0±0.3 | 68 | 5.7±0.6 |
| Weight loss subgroup^‡^: ≥5 – <10% | 41 | 5.8±0.3 | 17 | 5.8±0.4 | 40 | 5.3±0.3 | 17 | 5.6±0.4 | 35 | 5.7±0.3 | 15 | 5.7±0.4 |
| Weight loss subgroup^‡^: ≥10 – <15% | 44 | 5.9±0.3 | 5 | 5.8±0.3 | 45 | 5.3±0.3 | 5 | 5.4±0.3 | 36 | 5.7±0.3 | 5 | 5.6±0.2 |
| Weight loss subgroup^‡^: ≥15 – <20% | 48 | 5.7±0.3 | 3 | 5.5±0.2 | 48 | 5.2±0.2 | 3 | 5.3±.0.2 | 45 | 5.6±0.3 | 2 | 5.5±0.2 |
| Weight loss subgroup^‡^: ≥20% | 79 | 5.7±0.4 | 1 | 5.9±NA | 80 | 5.1±0.3 | 1 | 5.4±NA | 68 | 5.4±0.3 | 1 | 5.6±NA |
| Total cholesterol (mg/dL) |  |  |  |  |  |  |  |  |  |  |  |  |
| Weight loss subgroup^‡^: <5% | 14 | 185.3 (23.0) | 71 | 194.8 (20.0) | 14 | 192.7 (24.9) | 73 | 196.9 (19.1) | 12 | 196.7 (13.0) | 69 | 195.2 (20.2) |
| Weight loss subgroup^‡^: ≥5 – <10% | 41 | 191.6 (21.9) | 17 | 190.6 (17.7) | 41 | 188.0 (23.8) | 17 | 181.9 (21.2) | 35 | 185.5 (21.9) | 15 | 175.9 (19.1) |
| Weight loss subgroup^‡^: ≥10 – <15% | 44 | 194.3 (16.4) | 5 | 231.4 (13.0) | 45 | 184.3 (19.6) | 5 | 229.8 (16.1) | 36 | 193.8 (19.9) | 5 | 235.4 (13.7) |
| Weight loss subgroup^‡^: ≥15 – <20% | 48 | 205.4 (17.0) | 3 | 175.9 (10.0) | 48 | 198.2 (19.5) | 3 | 174.8 (19.6) | 44 | 203.0 (18.1) | 2 | 180.9 (0.2) |
| Weight loss subgroup^‡^: ≥20% | 80 | 188.4 (16.8) | 1 | 162.2 (0.0) | 80 | 174.0 (18.7) | 1 | 183.8 (0.0) | 68 | 185.0 (19.9) | 1 | 178.8 (0.0) |
| HDL cholesterol (mg/dL) |  |  |  |  |  |  |  |  |  |  |  |  |
| Weight loss subgroup^‡^: <5% | 14 | 45.2 (26.1) | 71 | 48.5 (28.5) | 14 | 46.8 (25.2) | 73 | 48.6 (27.1) | 12 | 48.4 (18.9) | 69 | 48.5 (25.8) |
| Weight loss subgroup^‡^: ≥5 – <10% | 41 | 49.9 (26.7) | 17 | 51.6 (23.5) | 41 | 52.3 (25.7) | 17 | 53.3 (24.0) | 35 | 50.7 (25.7) | 15 | 50.1 (26.0) |
| Weight loss subgroup^‡^: ≥10 – <15% | 44 | 48.9 (19.1) | 5 | 52.1 (14.0) | 45 | 50.9 (20.4) | 5 | 59.5 (15.2) | 35 | 49.0 (19.6) | 5 | 59.1 (14.1) |
| Weight loss subgroup^‡^: ≥15 – <20% | 48 | 51.3 (22.3) | 3 | 42.2 (17.0) | 48 | 54.2 (24.6) | 3 | 49.6 (13.4) | 43 | 52.7 (23.7) | 2 | 53.5 (1.5) |
| Weight loss subgroup^‡^: ≥20% | 80 | 48.7 (25.2) | 1 | 37.8  (0.0) | 80 | 54.5 (23.5) | 1 | 69.1  (0.0) | 68 | 57.8 (29.8) | 1 | 51.7 (0.0) |
| LDL cholesterol (mg/dL) |  |  |  |  |  |  |  |  |  |  |  |  |
| Weight loss subgroup^‡^: <5% | 14 | 100.36 (48.5) | 71 | 113.88 (26.9) | 14 | 106.70 (57.7) | 73 | 118.06 (27.5) | 12 | 107.61 (27.4) | 69 | 111.50 (28.6) |
| Weight loss subgroup^‡^: ≥5 – <10% | 41 | 110.62 (33.7) | 17 | 108.15 (29.0) | 41 | 108.32 (36.1) | 17 | 98.84 (52.1) | 35 | 104.82 (33.9) | 15 | 88.13 (48.5) |
| Weight loss subgroup^‡^: ≥10 – <15% | 44 | 114.61 (27.1) | 5 | 144.13 (15.7) | 45 | 107.84 (33.0) | 5 | 141.56 (14.1) | 35 | 109.12 (34.9) | 5 | 145.81 (23.6) |
| Weight loss subgroup^‡^: ≥15 – <20% | 48 | 122.87 (26.4) | 3 | 112.64 (12.7) | 48 | 120.06 (26.2) | 3 | 108.13 (25.9) | 44 | 119.04 (25.1) | 2 | 111.76 (2.7) |
| Weight loss subgroup^‡^: ≥20% | 80 | 111.29 (25.1) | 1 | 72.20 (0.0) | 80 | 102.12 (25.6) | 1 | 94.59 (0.0) | 68 | 103.88 (29.3) | 1 | 105.41 (0.0) |
| VLDL cholesterol (mg/dL) |  |  |  |  |  |  |  |  |  |  |  |  |
| Weight loss subgroup^‡^: <5% | 14 | 32.7 (25.4) | 71 | 25.5 (58.2) | 14 | 27.8 (49.2) | 73 | 24.0 (56.2) | 12 | 36.3 (30.2) | 69 | 27.9 (58.5) |
| Weight loss subgroup^‡^: ≥5 – <10% | 41 | 24.5 (53.4) | 17 | 26.4 (39.4) | 41 | 21.7 (51.3) | 17 | 22.4 (34.7) | 35 | 23.9 (53.5) | 15 | 28.8 (48.0) |
| Weight loss subgroup^‡^: ≥10 – <15% | 44 | 25.5 (47.9) | 5 | 31.6 (53.2) | 45 | 20.8 (45.3) | 5 | 24.4 (73.5) | 35 | 27.7 (49.8) | 5 | 24.4 (64.0) |
| Weight loss subgroup^‡^: ≥15 – <20% | 48 | 26.8 (41.8) | 3 | 20.5  (9.1) | 48 | 20.0 (43.8) | 3 | 16.5 (13.7) | 44 | 26.9 (43.3) | 2 | 15.5 (15.9) |
| Weight loss subgroup^‡^: ≥20% | 80 | 24.2 (41.6) | 1 | 52.1  (0.0) | 80 | 14.5 (39.3) | 1 | 20.1  (0.0) | 68 | 18.2 (49.0) | 1 | 21.6 (0.0) |
| Triglycerides (mg/dL) |  |  |  |  |  |  |  |  |  |  |  |  |
| Weight loss subgroup^‡^: <5% | 14 | 172.10 (33.8) | 71 | 130.33 (57.7) | 14 | 141.99 (49.1) | 73 | 122.28 (55.4) | 12 | 193.39 (40.3) | 69 | 143.46 (60.1) |
| Weight loss subgroup^‡^: ≥5 – <10% | 41 | 126.08  (55.4) | 17 | 135.25  (39.2) | 41 | 112.87 (56.2) | 17 | 114.47 (34.7) | 35 | 126.45  (61.0) | 15 | 147.40 (48.1) |
| Weight loss subgroup^‡^: ≥10 – <15% | 44 | 130.68 (48.6) | 5 | 161.70 (53.0) | 45 | 106.68 (45.2) | 5 | 124.48 (73.8) | 35 | 144.02 (53.8) | 5 | 124.69 (64.2) |
| Weight loss subgroup^‡^: ≥15 – <20% | 48 | 138.38 (45.5) | 3 | 104.72 (9.4) | 48 | 102.33  (43.9) | 3 | 84.23 (14.7) | 44 | 141.19 (51.0) | 2 | 79.20 (15.2) |
| Weight loss subgroup^‡^: ≥20% | 80 | 123.68 (41.6) | 1 | 267.00 (0.0) | 80 | 74.14 (39.3) | 1 | 102.35 (0.0) | 68 | 93.12 (48.9) | 1 | 111.25 (0.0) |
| C-reactive protein (mg/L) |  |  |  |  |  |  |  |  |  |  |  |  |
| Weight loss subgroup^‡^: <5% | 14 | 1.83 (214.0) | 72 | 2.96 (121.2) | 14 | 1.74 (295.5) | 73 | 3.08 (133.3) | 12 | 2.08 (186.0) | 69 | 2.75 (165.7) |
| Weight loss subgroup^‡^: ≥5 – <10% | 41 | 3.07 (181.1) | 17 | 3.29 (93.5) | 41 | 2.13 (217.3) | 17 | 2.06 (126.4) | 35 | 2.92 (159.7) | 16 | 2.31 (134.7) |
| Weight loss subgroup^‡^: ≥10 – <15% | 45 | 3.16  (170.9) | 5 | 5.02 (81.6) | 45 | 1.67 (177.7) | 5 | 2.47 (261.2) | 36 | 2.49 (186.5) | 5 | 2.93 (107.4) |
| Weight loss subgroup^‡^: ≥15 – <20% | 48 | 2.67 (163.2) | 3 | 3.67 (32.9) | 48 | 1.39 (177.6) | 3 | 1.32 (55.1) | 44 | 1.73 (128.0) | 2 | 2.71 (91.7) |
| Weight loss subgroup^‡^: ≥20% | 80 | 3.22 (162.8) | 1 | 0.83  (0.0) | 80 | 0.77 (190.5) | 1 | 0.18  (0.0) | 68 | 1.24 (206.6) | 1 | 1.03 (0.0) |

^†^Data are observed data presented as mean ± standard deviation or geometric mean (coefficient of variation) for the extension analysis set from the in-trial period.
^‡^Subgroup name indicates body weight loss from baseline to week 68.
HbA_1c_, glycated haemoglobin; HDL, high-density lipoprotein; LDL, low-density lipoprotein; NA, not applicable; VLDL, very-low-density lipoprotein.

## Table S6. Changes in cardiometabolic risk factors from baseline to week 120, by body weight loss from baseline to week 68 subgroups

|  | **Semaglutide arm** | | **Placebo arm** | |
| --- | --- | --- | --- | --- |
|  | **N** | **Change or ratio to baseline^†^** | **N** | **Change or ratio to baseline^†^** |
| Change from baseline to week 120 in systolic blood pressure (mmHg) |  |  |  |  |
| Weight loss subgroup^‡^: <5% | 12 | 12±14 | 69 | 2±15 |
| Weight loss subgroup^‡^: ≥5 – <10% | 35 | 1±14 | 16 | 4±17 |
| Weight loss subgroup^‡^: ≥10 – <15% | 37 | 6±16 | 5 | –1±4 |
| Weight loss subgroup^‡^: ≥15 – <20% | 45 | 1±12 | 2 | 9±10 |
| Weight loss subgroup^‡^: ≥20% | 68 | 1±13 | 1 | 1±NA |
| Change from baseline to week 120 in diastolic blood pressure (mmHg) |  |  |  |  |
| Weight loss subgroup^‡^: <5% | 12 | 5±11 | 69 | 1±10 |
| Weight loss subgroup^‡^: ≥5 – <10% | 35 | 0±9 | 16 | 4±9 |
| Weight loss subgroup^‡^: ≥10 – <15% | 37 | 3±12 | 5 | –3±4 |
| Weight loss subgroup^‡^: ≥15 – <20% | 45 | –1±11 | 2 | –2±10 |
| Weight loss subgroup^‡^: ≥20% | 68 | –1±9 | 1 | –3±NA |
| Change from baseline to week 120 in  HbA_1c_ (%) |  |  |  |  |
| Weight loss subgroup^‡^: <5% | 12 | 0.1±0.2 | 68 | 0.0±0.4 |
| Weight loss subgroup^‡^: ≥5 – <10% | 35 | –0.1±0.3 | 15 | –0.1±0.2 |
| Weight loss subgroup^‡^: ≥10 – <15% | 36 | –0.1±0.2 | 5 | –0.2±0.2 |
| Weight loss subgroup^‡^: ≥15 – <20% | 45 | –0.2±0.2 | 2 | –0.2±0.1 |
| Weight loss subgroup^‡^: ≥20% | 68 | –0.2±0.2 | 1 | –0.3±NA |
| Ratio from baseline to week 120 in total cholesterol |  |  |  |  |
| Weight loss subgroup^‡^: <5% | 12 | 1.04 (15.5) | 67 | 1.01(16.9) |
| Weight loss subgroup^‡^: ≥5 – <10% | 35 | 0.98 (15.0) | 15 | 0.91 (25.2) |
| Weight loss subgroup^‡^: ≥10 – <15% | 36 | 1.00 (10.6) | 5 | 1.02 (10.0) |
| Weight loss subgroup^‡^: ≥15 – <20% | 44 | 0.99 (12.1) | 2 | 1.02 (14.3) |
| Weight loss subgroup^‡^: ≥20% | 68 | 0.99 (11.4) | 1 | 1.10 (0.0) |
| Ratio from baseline to week 120 in HDL cholesterol |  |  |  |  |
| Weight loss subgroup^‡^: <5% | 12 | 1.00 (11.1) | 67 | 1.00 (18.0) |
| Weight loss subgroup^‡^: ≥5 – <10% | 35 | 1.02 (14.2) | 15 | 0.98 (7.2) |
| Weight loss subgroup^‡^: ≥10 – <15% | 35 | 1.01 (12.4) | 5 | 1.13 (11.6) |
| Weight loss subgroup^‡^: ≥15 – <20% | 43 | 1.04 (13.1) | 2 | 1.37 (17.0) |
| Weight loss subgroup^‡^: ≥20% | 68 | 1.18 (18.8) | 1 | 1.37 (0.0) |
| Ratio from baseline to week 120 in LDL cholesterol |  |  |  |  |
| Weight loss subgroup^‡^: <5% | 12 | 1.03 (27.0) | 67 | 0.99 (29.1) |
| Weight loss subgroup^‡^: ≥5 – <10% | 35 | 0.98 (27.1) | 15 | 0.81 (55.1) |
| Weight loss subgroup^‡^: ≥10 – <15% | 35 | 0.96 (18.1) | 5 | 1.01 (16.1) |
| Weight loss subgroup^‡^: ≥15 – <20% | 44 | 0.97 (18.9) | 2 | 0.97 (14.1) |
| Weight loss subgroup^‡^: ≥20% | 68 | 0.95 (16.0) | 1 | 1.46 (0.0) |
| Ratio from baseline to week 120 in VLDL cholesterol |  |  |  |  |
| Weight loss subgroup^‡^: <5% | 12 | 1.16 (21.5) | 67 | 1.08 (41.1) |
| Weight loss subgroup^‡^: ≥5 – <10% | 35 | 0.97 (42.5) | 15 | 1.07 (41.0) |
| Weight loss subgroup^‡^: ≥10 – <15% | 35 | 1.09 (33.8) | 5 | 0.77 (32.0) |
| Weight loss subgroup^‡^: ≥15 – <20% | 44 | 0.98 (39.5) | 2 | 0.72 (13.3) |
| Weight loss subgroup^‡^: ≥20% | 68 | 0.76 (45.3) | 1 | 0.41 (0.0) |
| Ratio from baseline to week 120 in triglycerides |  |  |  |  |
| Weight loss subgroup^‡^: <5% | 12 | 1.21 (30.1) | 67 | 1.09 (42.5) |
| Weight loss subgroup^‡^: ≥5 – <10% | 35 | 0.99 (44.0) | 15 | 1.06 (41.3) |
| Weight loss subgroup^‡^: ≥10 – <15% | 35 | 1.11 (39.9) | 5 | 0.77 (32.7) |
| Weight loss subgroup^‡^: ≥15 – <20% | 44 | 1.00 (40.9) | 2 | 0.72 (11.7) |
| Weight loss subgroup^‡^: ≥20% | 68 | 0.76 (45.4) | 1 | 0.42 (0.0) |
| Ratio from baseline to week 120 in C-reactive protein |  |  |  |  |
| Weight loss subgroup^‡^: <5% | 12 | 1.33 (90.8) | 68 | 0.97 (94.1) |
| Weight loss subgroup^‡^: ≥5 – <10% | 35 | 0.99 (92.2) | 16 | 0.72 (56.1) |
| Weight loss subgroup^‡^: ≥10 – <15% | 36 | 0.82 (94.4) | 5 | 0.58 (67.8) |
| Weight loss subgroup^‡^: ≥15 – <20% | 44 | 0.66 (80.2) | 2 | 0.83 (43.6) |
| Weight loss subgroup^‡^: ≥20% | 68 | 0.42 (119.4) | 1 | 1.24 (0.0) |

**^†^**Data are observed data presented as mean change from baseline ± standard deviation or geometric mean ratio to baseline (coefficient of variation) for the extension analysis set from the in-trial period.
^‡^Subgroup name indicates body weight loss from baseline to week 68.
HbA_1c_, glycated haemoglobin; HDL, high-density lipoprotein; LDL, low-density lipoprotein; NA, not applicable; VLDL, very-low-density lipoprotein.

# Supplementary figures

## Figure S1. Trial design


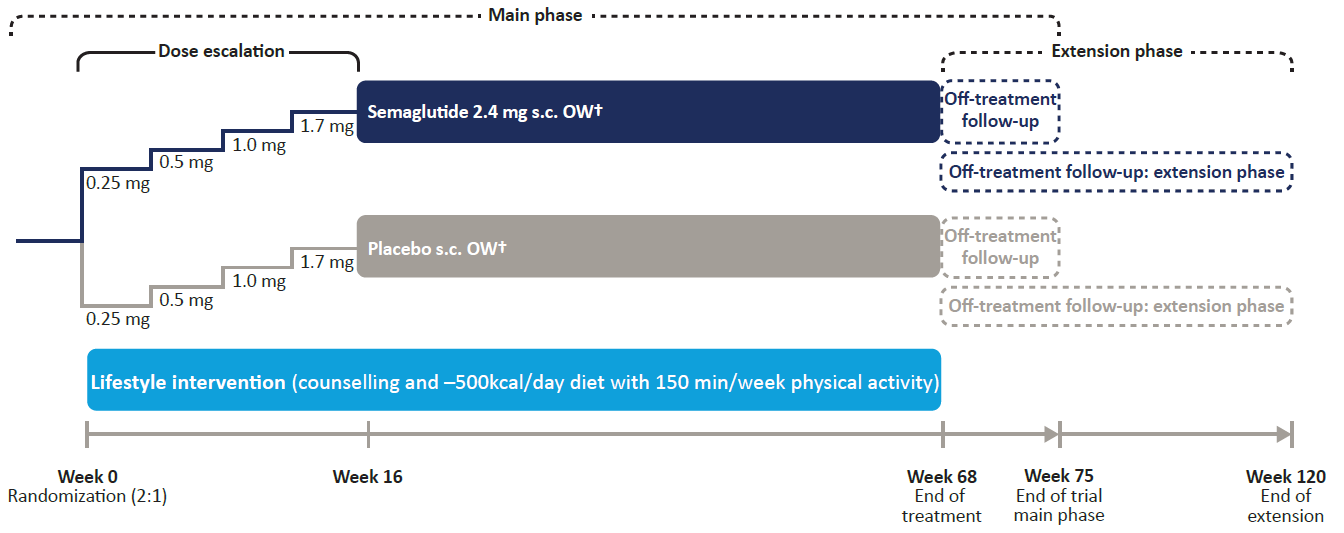


^†^As an adjunct to lifestyle intervention (–500 kcal/day diet with 150 min/week physical activity).
Figure S1 is adapted from N Engl J Med, John P.H. Wilding, Rachel L. Batterham, Salvatore Calanna et al., Once-weekly semaglutide in adults with overweight or obesity, 384: 989–1002 (1). Copyright © 2021 Massachusetts Medical Society. Reprinted with permission from Massachusetts Medical Society.
OW, once weekly; s.c., subcutaneous.

## Figure S2. Participant flow


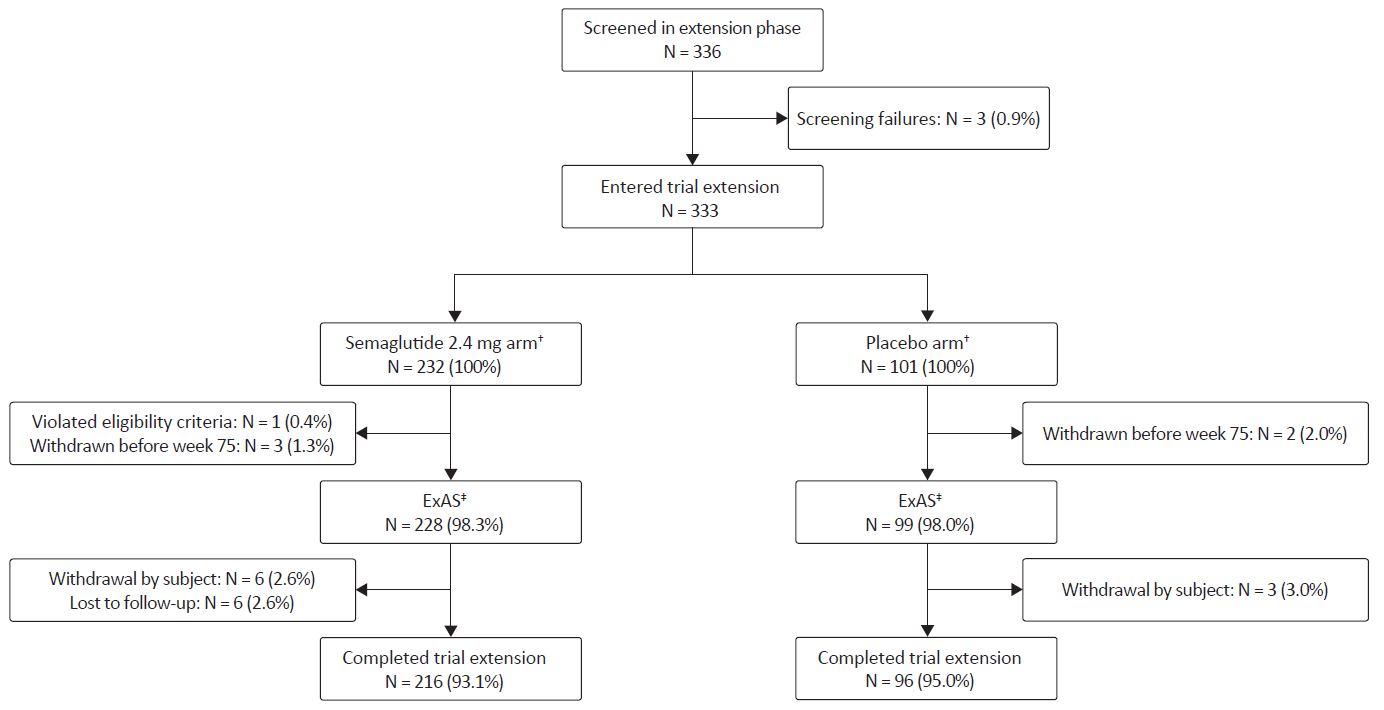


^†^Treatment groupings based on randomized allocation in the main phase of the trial.
^‡^ExAS includes all participants eligible for the extension trial, who gave informed consent to participate and attended at least one of the four visits between week 75 and week 120.
ExAS, extension analysis set.

## Figure S3. Lipid levels by week


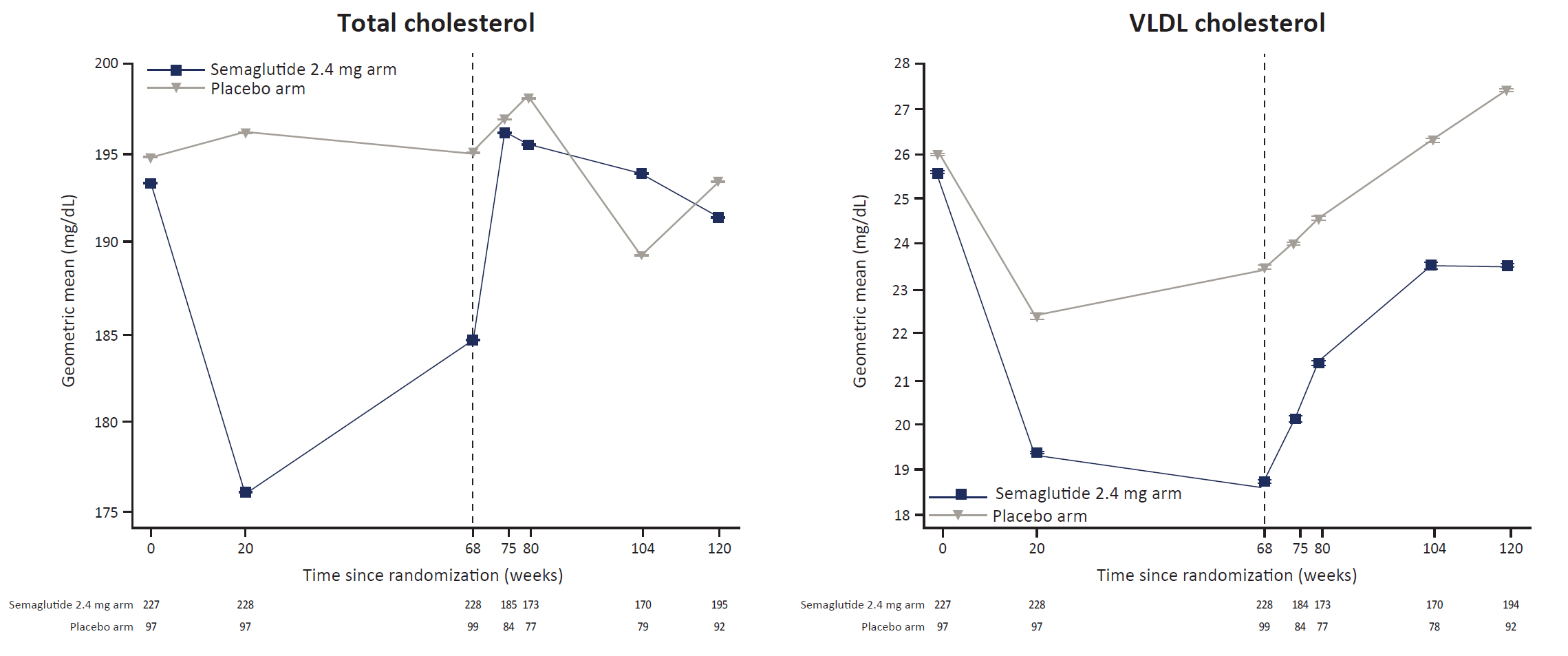


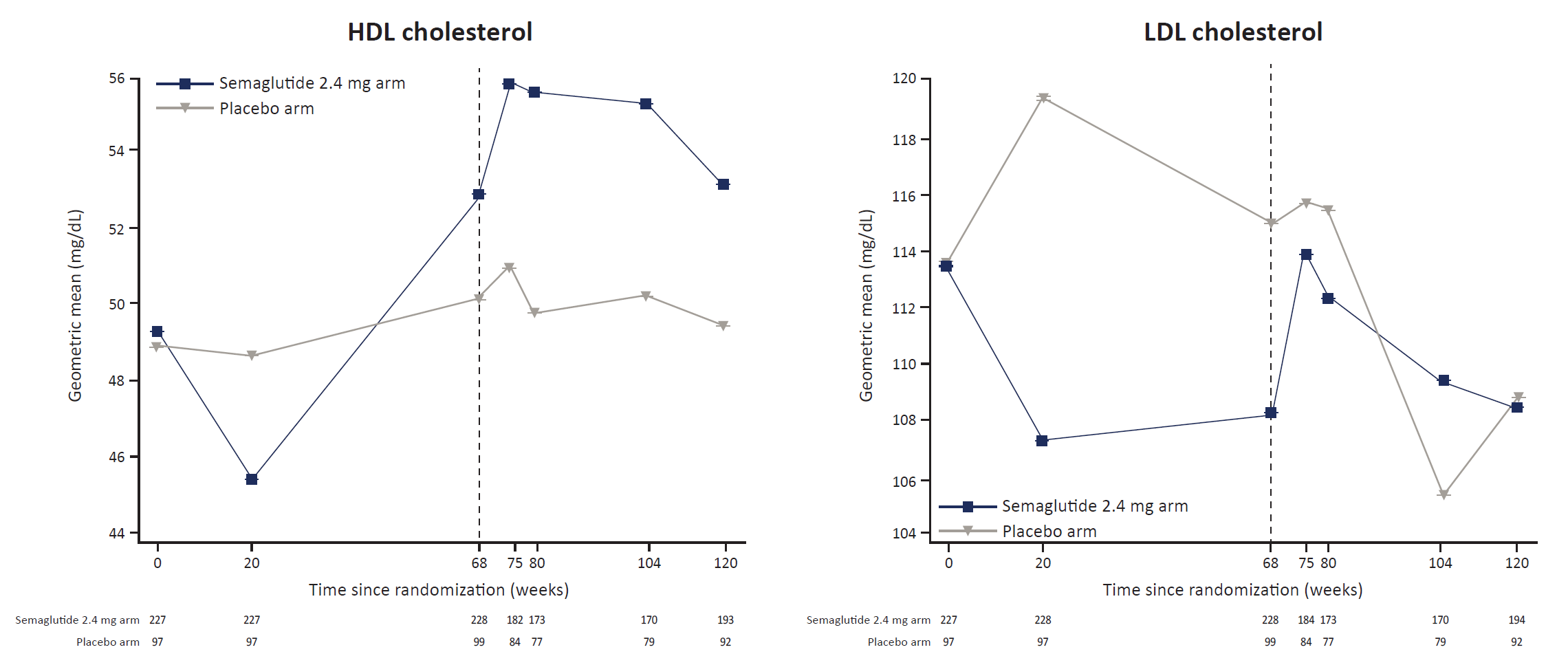


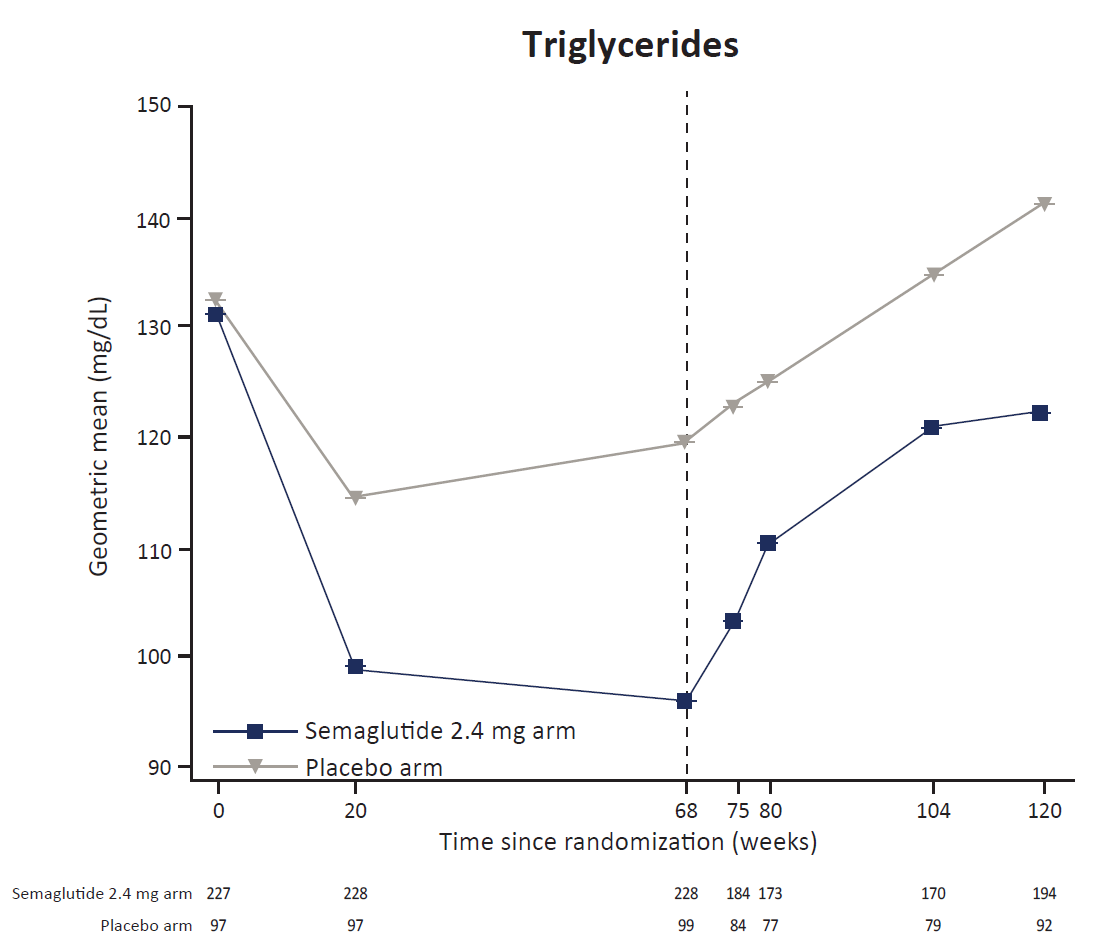


Data are observed means (± standard error calculated on the logarithmic scale and back-transformed to the linear scale) for the extension analysis set from the in-trial period. The dashed vertical lines at week 68 indicate the end of the main phase and start of the off-treatment extension phase. Numbers shown in the lower panels are participants contributing to the mean. Free fatty acids are not shown due to different fasting requirements in the main phase and the extension phase.
HDL, high-density lipoprotein; LDL, low-density lipoprotein; VLDL, very-low-density lipoprotein.

# Supplementary references

1. American Diabetes Association. 2. Classification and diagnosis of diabetes. *Diabetes Care*. 2017;40(Suppl 1):S11-S24.
